# FLASH radiotherapy enables dose escalation resulting in improved survival in an orthotopic muscle-invasive bladder cancer mouse model

**DOI:** 10.1093/bjr/tqag071

**Published:** 2026-03-26

**Authors:** Jia-Ling Ruan, Carl Lee, Osheen Sharma, Nathalie Lövgren, Salomé Paillas, Christian Cooper, Iain D C Tullis, Amato J Giaccia, Anne E Kiltie, Kristoffer Petersson

**Affiliations:** Department of Oncology, University of Oxford, Oxford, OX3 7DQ, United Kingdom; Kennedy Institute of Rheumatology, University of Oxford, Oxford, OX3 7FY, United Kingdom; Nuffield Department of Medicine, University of Oxford, Oxford, OX3 7BN, United Kingdom; Department of Oncology, University of Oxford, Oxford, OX3 7DQ, United Kingdom; Department of Oncology, University of Oxford, Oxford, OX3 7DQ, United Kingdom; Department of Oncology, University of Oxford, Oxford, OX3 7DQ, United Kingdom; Department of Oncology, University of Oxford, Oxford, OX3 7DQ, United Kingdom; Department of Oncology, University of Oxford, Oxford, OX3 7DQ, United Kingdom; Department of Oncology, University of Oxford, Oxford, OX3 7DQ, United Kingdom; The Rowett Institute, School of Medicine, Medical Sciences and Nutrition, University of Aberdeen, Aberdeen, AB25 2ZD, United Kingdom; Aberdeen Cancer Centre, University of Aberdeen, Aberdeen, Aberdeen, AB25 2ZN, United Kingdom; Department of Oncology, University of Oxford, Oxford, OX3 7DQ, United Kingdom; Department of Haematology, Oncology and Radiation Physics, Skåne University Hospital, Lund University, Lund, 221 85, Sweden

**Keywords:** FLASH irradiation, muscle-invasive bladder cancer, radiotherapy, normal tissue toxicity, dose rate, orthotopic cancer models

## Abstract

**Objectives:**

FLASH radiotherapy is an innovative technique that delivers radiation at ultra-high dose rates (UHDR), offering tumor control comparable to conventional (CONV) radiotherapy while significantly reducing normal tissue toxicity. Here we aim to determine the effects of FLASH compared to CONV radiotherapy in muscle-invasive bladder cancer (MIBC) models.

**Methods:**

Using an in-house 6 MeV linear accelerator able to deliver electron beam at UHDR or CONV dose rate, we employed clonogenic survival assays, RNA sequencing (RNA-seq), and *in vivo* tumor growth analyses using MBT2 cells and C3H MIBC models. Both subcutaneous and orthotopic tumor models were used to assess tumor response, survival and treatment-related toxicity as demonstrated by weight loss.

**Results:**

Clonogenic analysis demonstrated comparable cancer cell survival between FLASH and CONV irradiation *in vitro*. RNA-seq analysis of *in vitro* irradiated cells revealed similar gene expression at 5 Gy but significant transcriptional divergence at 10 Gy. Intestinal organoids exhibited preserved growth after FLASH compared with CONV irradiation, consistent with a normal tissue sparing effect. In subcutaneous models, FLASH and CONV radiotherapy exhibited similar tumor responses. However, in the orthotopic model, FLASH radiotherapy enabled dose escalation, significantly extending survival at 15 Gy (*P = .*02) and 17.5 Gy (*P = .*004). Dose rate (100 vs. 10^6^ Gy/s) did not significantly affect survival. The benefit of single-fraction FLASH was not retained with fractionated (3 × 7.3 Gy) delivery.

**Conclusions:**

FLASH radiotherapy demonstrates significant potential for treating MIBC, offering enhanced survival through effective dose escalation. These findings support continued investigation into optimal FLASH parameters and its clinical application.

**Advances in knowledge:**

This study demonstrates, for the first time, that UHDR radiotherapy enables safe dose escalation and improved survival in an orthotopic muscle-invasive bladder cancer model compared to conventional dose-rate treatment. Survival benefits of FLASH are dose- and fractionation-dependent, with significant improvements observed in single-fraction, high-dose treatments but not in fractionated delivery.

## Introduction

Muscle-invasive bladder cancer (MIBC), characterized by cancer invasion into the bladder wall detrusor muscle, accounts for around 20%-25% of new bladder cancer cases, with a 5-year survival of ∼50% despite good local control.^1^ Up to 50% of non-MIBC cases (stage Ta/T1) will progress to MIBC.^2^ While cystectomy remains a standard of care treatment,^3^ it significantly impacts patients’ quality of life. Bladder-preserving strategies including neo-adjuvant chemotherapy, radiotherapy (RT) and chemoradiation^4–6^ offer curative potential with organ preservation.^7^^,^^8^ However, surrounding normal tissue is inevitably irradiated and ∼30%-40% of MIBC patients experience acute toxicity,^9^^,^^10^ including diarrhea as a common early side effect.^11^

FLASH RT, a novel ultra-high dose rate technique,^12^ shows promise in preclinical and veterinary studies by reducing normal tissue toxicity while preserving tumor control.^13^^,^^14^ Our previous work and others’ have shown FLASH RT mitigates gastrointestinal toxicity and improve survival in mice compared to conventional dose rate (CONV) RT.^15–18^

Recently, several early-phase clinical trials have begun translating ultra-high dose rate (UHDR) or FLASH RT into patient treatment settings. The first-in-human proton FLASH trial (FAST-01, NCT04592887) demonstrated feasibility and safety in patients with bone metastases, followed by the ongoing FAST-02 study (NCT05524064) assessing thoracic bone lesions.^19^^,^^20^ In parallel, several electron-based FLASH trials, including IMPULSE (NCT04986696), Flash-Skin 1 (NCT06549439), LANCE (NCT05724875), and a recently initiated Phase I study (ChiCTR2400080935), are evaluating the safety and clinical efficacy of UHDR electron FLASH RT for superficial skin tumors.^21–23^ Together, these studies mark a key step toward clinical translation of FLASH RT and highlight the expanding interest in assessing its normal-tissue-sparing potential in humans.

In this study, we compared MBT2 tumor response following FLASH and CONV RT. We first investigated the effect on *in vitro* 2D cell models, followed by *in vivo* subcutaneous and orthotopic tumor models of MIBC, both established in C3H mice inoculated with MBT2 transitional bladder cancer cells. We also evaluated how variations in FLASH delivery regarding dose rate used and single versus hypofractionated treatment influence outcome in the orthotopic MIBC model, offering a broader insight into the therapeutic potential of FLASH RT.

## Methods

All animal work was performed by PIL (Personal Licence) holders in accordance with UK Home Office Guidelines and institutional guidelines, under University of Oxford project licenses PPL PP8415318 and P8484EDAE.

Detailed protocol for IR setup, dose fractionation parameters, dosimetry, RNA-seq, and clonogenic assay are in the Supplementary Document.

### Radiation setup

A custom 6 MeV electron linear accelerator was used to deliver a horizontal beamline at CONV and UHDR settings for CONV and FLASH RT, respectively.^24^

For subcutaneous tumors, the mice were placed upright behind a 6-mm brass collimator with a 14-mm circular central aperture, aligning the beam with the tumor. For orthotopic tumors, mice were inverted to displace the intestines from the field^25^ and positioned behind a similar 6 mm brass collimator but with a 15 × 30 mm^2^ (*h* × *w*) rectangular aperture targeting the lower abdomen (Figure S1).

*In vitro* irradiations were performed in air and at room temperature (21% oxygen, and 20 °C), corresponding to normoxia. During irradiation, all mice were maintained at 2% isoflurane in 1.5 L/min supplemented with ≈55% oxygen (≈95% oxygen mixed with air, 1:1 ratio) with less than 10 min total anesthetic time.

CONV treatment was delivered with numerous (≈2000-4400) 3.4 µs electron pulses, at 25 Hz pulse repetition frequency, for an average dose rate of ≈0.1 Gy/s. FLASH treatment was delivered with 1-4 (3.4 µs) pulses of 2.5 (organoids only) or 5 Gy, for the *in vitro* and subcutaneous tumor treatments, and 2-4 pulses of ≈5 Gy (2, 3, 3, 3, and 4 pulses for a 10, 12.5, 15, 17.5, and 20 Gy delivery, respectively) for the orthotopic tumor treatments. The pulse repetition frequency was 300 Hz, yielding average dose rates ≥2000 Gy/s.

### Cells and materials

MBT2 cells were kindly provided by Prof Peter Black (University of British Columbia) and tested negative for mycoplasma. It was originally derived from a carcinogen-induced transitional cell carcinoma that developed in a C3H/He mouse following N-butyl-N-(4-hydroxybutyl) nitrosamine (BBN) exposure.^26^ It is a moderately to highly invasive murine urothelial carcinoma line that forms orthotopic tumors recapitulating key features of human muscle-invasive bladder cancer, including detrusor muscle infiltration and local invasion. MBT2 cells are p53 wild type but harbor Ras pathway activation consistent with their chemically induced origin and have been widely used as an immunocompetent bladder cancer model for preclinical research. Cells were cultured in RPMI medium with 10% FBS, 1% penicillin/streptomycin (100 U/mL) and maintained at 37 °C in a humidified incubator with 5% CO_2_. Cells for *in vivo* injection were mixed 1:1 with high concentration Matrigel (Corning 354262).

### Intestinal organoid culture and quantification

Murine small-intestinal organoids were generated from 3 biologically independent preparations derived from adult female C3H mice. Small intestines were harvested, flushed with cold PBS, opened longitudinally, and villi were removed by gentle scraping. Tissue fragments were incubated in EDTA-based dissociation buffer to release intestinal crypts, which were subsequently collected by mechanical shaking and filtration. Intestinal crypts were embedded in Matrigel domes (Corning 356231) and maintained in IntestiCult organoid growth medium (STEMCELL Technologies) according to the manufacturer’s protocol. Organoids were cultured for 7 days and passaged once prior to irradiation to allow formation of mature spheroid structures. For irradiation experiments, organoids were plated onto 3.5 cm dishes (µ-Dish 35 mm, high; iBidi) at approximately 50 crypts per 50 µL Matrigel dome and allowed to recover overnight before treatment.

Bright-field images were acquired on day 4 post-irradiation using an inverted microscope (Nikon TiE) under identical magnification and exposure settings for all experimental groups. Images were calibrated using the microscope scale bar, and measurements were performed in ImageJ/Fiji.

Organoid growth was quantified by measuring the 2-dimensional cross-sectional area (µm^2^) of individual organoids from bright-field images. Organoids were manually thresholded to delineate borders, and the projected area was automatically calculated by ImageJ using pixel-to-micrometre conversion. Only intact, clearly delineated organoids were included in the analysis; collapsed or fragmented structures were excluded. Cross-sectional area was used as a surrogate measure of organoid growth and viability. For each biological replicate, at least 100 organoids per condition were analyzed and pooled for statistical comparison.

### Establishment of subcutaneous bladder cancer models

Forty-two female C3H mice (7-8 weeks old, with an average weight of 20.7 ± 0.2 g) were subcutaneously injected with 10^6^ MBT2 cells in the right flank. Ten days post-tumor inoculation (tumor size = 104 ± 5 mm^3^) the mice were randomly allocated to different groups for either CONV or FLASH RT. Tumor size was monitored using calipers, and mice were euthanized when the tumor size reached 700 mm^3^. Tumor volume was calculated using the equation: Volume = Length × Width × Height  ×  π/6.

### Establishment of orthotopic bladder cancer

The orthotopic MIBC model was established using ultrasound-guided intramural injection as described and illustrated by Jäger et al and was reproduced here with minor modifications.^27^^,^^28^ Female C3H mice (10-14 weeks old, average weight: 24.4 ± 0.4 g) were used because their shorter and straighter urethra allows easier intravesical catheterization and improved ultrasound visualization of the bladder. Mice were anesthetized with 4% isoflurane and positioned supine on a heated imaging platform. Under real-time micro-ultrasound guidance (Vevo 3100, Fujifilm), the anterior bladder wall was first expanded by injecting 50 µL of sterile phosphate-buffered saline (PBS) into the submucosal layer to create a clear separation between the mucosa and muscularis. This PBS pre-injection formed an artificial pocket that facilitated accurate subsequent tumor-cell placement. A 30-gauge needle was then inserted through the bladder dome, and 20 µL of a suspension containing 1 × 10^6^ MBT2 cells mixed 1:1 with high-concentration Matrigel (Corning 354262) was injected into this space. Correct intramural deposition was confirmed by the formation of a well-defined hypoechoic bleb on ultrasound.

Seven to 10 days after tumor inoculation (tumor size = 78.5 ± 4.7 mm^3^), mice were randomly assigned to different groups for CONV or FLASH RT. The tumor size was measured weekly using micro-ultrasound imaging (Vevo3100, Fujifilm, Toronto, Canada). Mice were checked daily and weighed at least twice weekly. Humane endpoints were predefined in accordance with UK Home Office license requirements. Mice were euthanized if the tumor reached 450 mm³, if body weight decreased by more than 15% from pre-treatment baseline, or if signs of treatment-related toxicity or distress were observed. These included severe or persistent hematuria, hunched posture, lethargy, reduced mobility, abdominal discomfort, or other sustained clinical signs indicating compromised welfare in the absence of tumor progression.

### Statistical analysis

Data are presented as mean ± SEM. For subcutaneous tumors, tumor progression was analyzed using time-to-tumor-tripling (TTT) rather than mean tumor-volume curves, as the MBT2 subcutaneous model exhibits heterogeneous growth dynamics and all mice eventually reached the tripling endpoint. TTT provides a consistent and statistically robust time-to-event metric comparable to the humane-endpoint analysis used in the orthotopic model. In the orthotopic model, survival was time to euthanasia per above criteria. Survival data were analyzed using the Log-rank test. *t*-tests compared 2 groups; 1-/2-way ANOVA was used for multiple comparisons (Tukey’s or Fisher’s test). Significance was set at *P < .*05.

## Results

### Effect of CONV and FLASH IR *in vitro*

Clonogenic analysis demonstrated similar cell survival following CONV (≈0.1 Gy/s) or FLASH (≥2000 Gy/s) IR (Figure S2). Survival curves were fitted using a weighted linear-quadratic model (*R*^2^: CONV = 0.9764; FLASH = 0.9853). CONV IR yielded a higher α/β ratio (1384; α = .3505, 95% CI: 0.2413-0.4215; β = .0002533, 95% CI: −0.004195 to 0.006026) than FLASH IR (178.5; α = .3136, 95% CI: 0.2363-0.3711; β = .001757, 95% CI: −0.001918 to 0.006045). Although the best-fit α/β estimates differed numerically between CONV (1384) and FLASH (178.5), the β terms for both fits had wide confidence intervals crossing zero, indicating that the curvature is poorly constrained and that both dose-response relationships are compatible with an approximately exponential decline. The 95% confidence intervals for α and β overlapped substantially between modalities, indicating no statistically meaningful difference in radiosensitivity *in vitro*.

**Figure 1:**
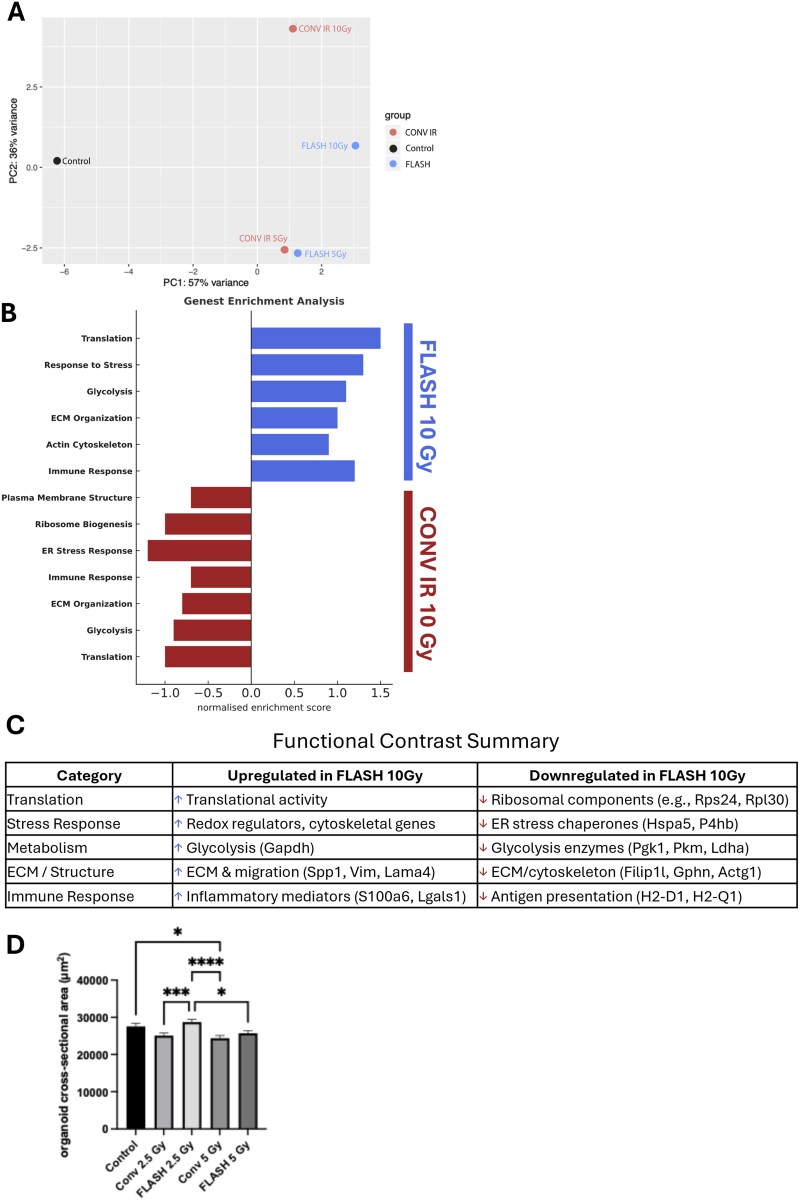
Similar clonogenic survival following CONV or FLASH IR *in vitro* but with divergent gene enrichment following 10 Gy IR. (A) Principal component analysis (PCA) of RNA-seq data from pooled biological triplicates irradiated with 0, 5, or 10 Gy at CONV or FLASH dose rates, showing clear separation of samples by dose and irradiation modality. Each data point represents one pooled sample per treatment condition. (B) Gene ontology (GO) enrichment analysis of differentially expressed genes following 10 Gy exposure, highlighting pathways upregulated in FLASH 10 Gy (blue) and CONV 10 Gy (red). (C) Functional contrast summary comparing GO biological processes enriched in cells exposed to 10 Gy FLASH versus CONV irradiation. (D) Normal tissue sparing in intestinal organoids following FLASH versus CONV irradiation. Murine intestinal organoids generated from 3 biologically independent preparations were exposed to CONV or FLASH irradiation at 2.5 Gy or 5 Gy, and the cross-sectional area of organoid was quantified on day 4 post-irradiation. FLASH-irradiated organoids retained markedly larger sizes than those treated with CONV irradiation, particularly at 5 Gy, indicating enhanced preservation of epithelial growth capacity. Data are presented as individual organoid measurements with group medians. Statistical analysis was performed using Kruskal-Wallis with Dunn’s *post hoc* test.

Principal component analysis (PCA) of RNA-seq data (24 hours post-IR) revealed that the first principal component (PC1, 57% variance) separates irradiated from control samples, indicating a strong transcriptional response to radiation, while PC2 (36% variance) reflects a dose-dependent effect (Figure 1A). Gene expression profiles were similar between CONV and FLASH IR of 5 Gy, but a more pronounced transcriptional divergence emerged at 10 Gy between the 2 IR modalities. To visualize dose-dependent transcriptional divergence between FLASH and CONV irradiation, side-by-side volcano plots were generated for the 5 Gy and 10 Gy comparisons (Figure S3A). The 10 Gy volcano plot showed a substantial number of differentially expressed genes, with numerous transcripts meeting |log_2_FC| > 1 and FDR < 0.05, whereas the 5 Gy comparison showed only a small number of significant differentially expressed genes (DEGs). This supports a clear dose-dependent divergence in transcriptional response between FLASH and CONV irradiation. A focused heatmap of biologically relevant genes further illustrated this divergence at 10 Gy, showing clear separation between FLASH- and CONV-treated samples (Figure S3B). At 5 Gy, no pathways reached conventional statistical significance, although several top-ranked gene sets, primarily related to stress response, ion transport and innate immune regulation, showed directional enrichment patterns that were consistent in sign with the 10 Gy comparisons (Figure S3C). Given this dose-dependent overview, the subsequent analyses focus on the statistically significant pathways at 10 Gy and the associated gene-level contrasts.

Gene enrichment analysis revealed sharply contrasting biological responses to FLASH 10 Gy versus CONV 10 Gy IR (Figure 1B). Pathway enrichment significance was determined using GSEA-derived false discovery rate (FDR) values, which are calculated by permutation and are independent of replicate variance. FLASH treatment was associated with high normalized enrichment scores (NES = 0.8-1.4, FDR < 0.05) in key functional pathways, including translation, stress response, glycolysis, extracellular matrix (ECM) organization, actin cytoskeleton remodeling and immune response (Figure 1B). This indicates an overall activation of processes that support cellular adaptation, structural reorganization and metabolic resilience. Conversely, CONV 10 Gy shows lower NES values (−0.7 to −1.2, FDR < 0.05) in a broad range of pathways; plasma membrane structure, ribosome biogenesis, endoplasmic reticulum (ER) stress response, immune function, ECM organization, glycolysis, and translation.

In response to 10 Gy FLASH, distinct transcriptional changes are observed across multiple biological categories (Figure 1C). In the category of translation, there is an apparent paradox where translational activity is upregulated yet core ribosomal components such as *Rps24* and *Rpl30* are downregulated, suggesting a shift in translational regulation or efficiency. The stress response profile shows an upregulation of redox regulators and cytoskeletal genes, which may reflect a compensatory response to radiation-induced stress, while classical ER stress chaperones like *Hspa5* and *P4hb* are reduced, possibly reflecting a distinct stress-handling mechanism under FLASH conditions compared to CONV. Metabolic shifts include increased expression of *Gapdh*, associated with glycolysis, whereas other glycolytic enzymes like *Pgk1*, *Pkm* and *Ldha* are downregulated, implying a noncanonical or incomplete glycolytic flux. Within the ECM and structural organization, genes related to ECM remodeling and cell migration such as *Spp1*, *Vim* and *Lama4* are elevated, whereas components linked to structural integrity and cytoskeletal function like *Filip1l*, *Gphn* and *Actg1* are diminished, suggesting active tissue remodeling. Finally, in the immune response, pro-inflammatory mediators such as *S100a6* and *Lgals1* are upregulated, while key antigen presentation molecules *H2-D1* and *H2-Q1* are downregulated. These transcriptional shifts point to immune and metabolic alterations following FLASH IR, though the biological consequences, particularly in a tissue context, remain to be further validated.

To assess normal-tissue sensitivity, murine intestinal organoids were irradiated with CONV or FLASH dose rates, and organoid size was measured on day 4 post-irradiation. Organoid size differed significantly among groups (Kruskal-Wallis *H* = 28.38, *P < .*0001). Dunn’s multiple-comparison test showed that organoid growth was significantly reduced after CONV 5 Gy compared with control (*P = .*0315), whereas FLASH 5 Gy did not differ from control (*P > .*9999). FLASH-treated organoids also remained significantly larger than those exposed to CONV 2.5 Gy (*P = .*0007) and CONV 5 Gy (*P < .0001). These findings demonstrate a clear sparing effect of FLASH irradiation on intestinal organoid growth (Figure 1D), consistent with improved normal-tissue tolerance at UHDR.

### No significant difference in tumor response following CONV and FLASH RT in a subcutaneous MIBC model

**Figure 2:**
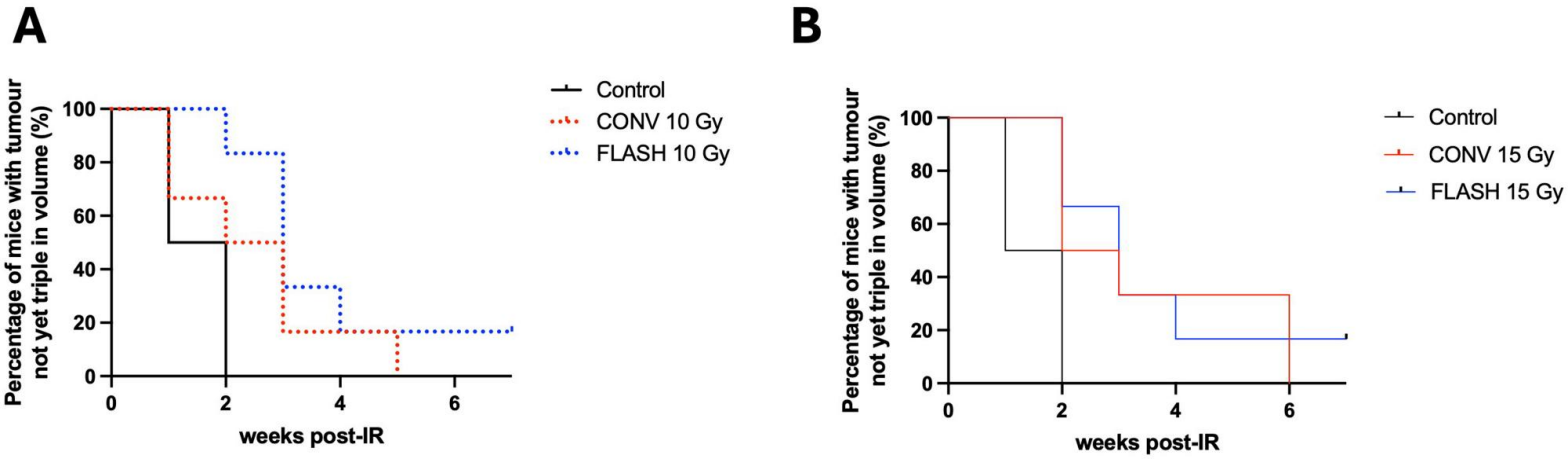
Comparable tumor growth delay following FLASH and CONV RT in MBT2 tumors. Time-to-tumor tripling curves for subcutaneous MBT2 tumors in C3H mice treated with 10 Gy (A) or 15 Gy (B) FLASH or CONV RT. No significant differences were observed between FLASH and CONV RT at either dose. *N* = 6 per group.

Subcutaneous tumors in mice were treated with 10 or 15 Gy CONV RT, or 5, 10, 15, or 20 Gy FLASH RT (Figure 2 and Figure S4). Tumors in the control group progressed rapidly, with median time to tripling at 1.5 weeks and all tumors tripled by week 2. Treatment with 10 or 15 Gy CONV RT delayed tripling to a median of 2.5 weeks, but no tumors remained controlled beyond week 6. FLASH RT at 5 Gy modestly extended tripling time to 2 weeks. Higher FLASH doses (10, 15, and 20 Gy) delayed tumor growth further, with a median time to tripling of 3 weeks. Notably, long-term tumor control, defined as tumors not tripled by the 7-week endpoint, was observed only in the FLASH 10 and 15 Gy groups, with one tumor in each group not tripling in size within 7 weeks (Figure 2). Despite this trend, there were no statistically significant differences between CONV and FLASH RT at 10 Gy (*P = .*27) or 15 Gy (*P = .*77) (Figure 2). Compared to controls, significant tumor growth delay was observed with both 10 (*P = .*003) and 15 Gy (*P = .*0003) CONV RT, as well as with FLASH RT at 10 (*P < .*0001), 15 (*P = .*008), and 20 Gy (*P = .*002). FLASH 5 Gy did not significantly differ from controls (*P = .*8) (Figure S4).

### FLASH RT enables dose escalation and prolonged survival for an orthotopic MIBC model

Survival was determined based on tumor size reaching 450 mm³ or signs of toxicity-related side effects (>15% weight loss, severe hematuria, or severe distress such as hunched posture and lethargy).

**Figure 3:**
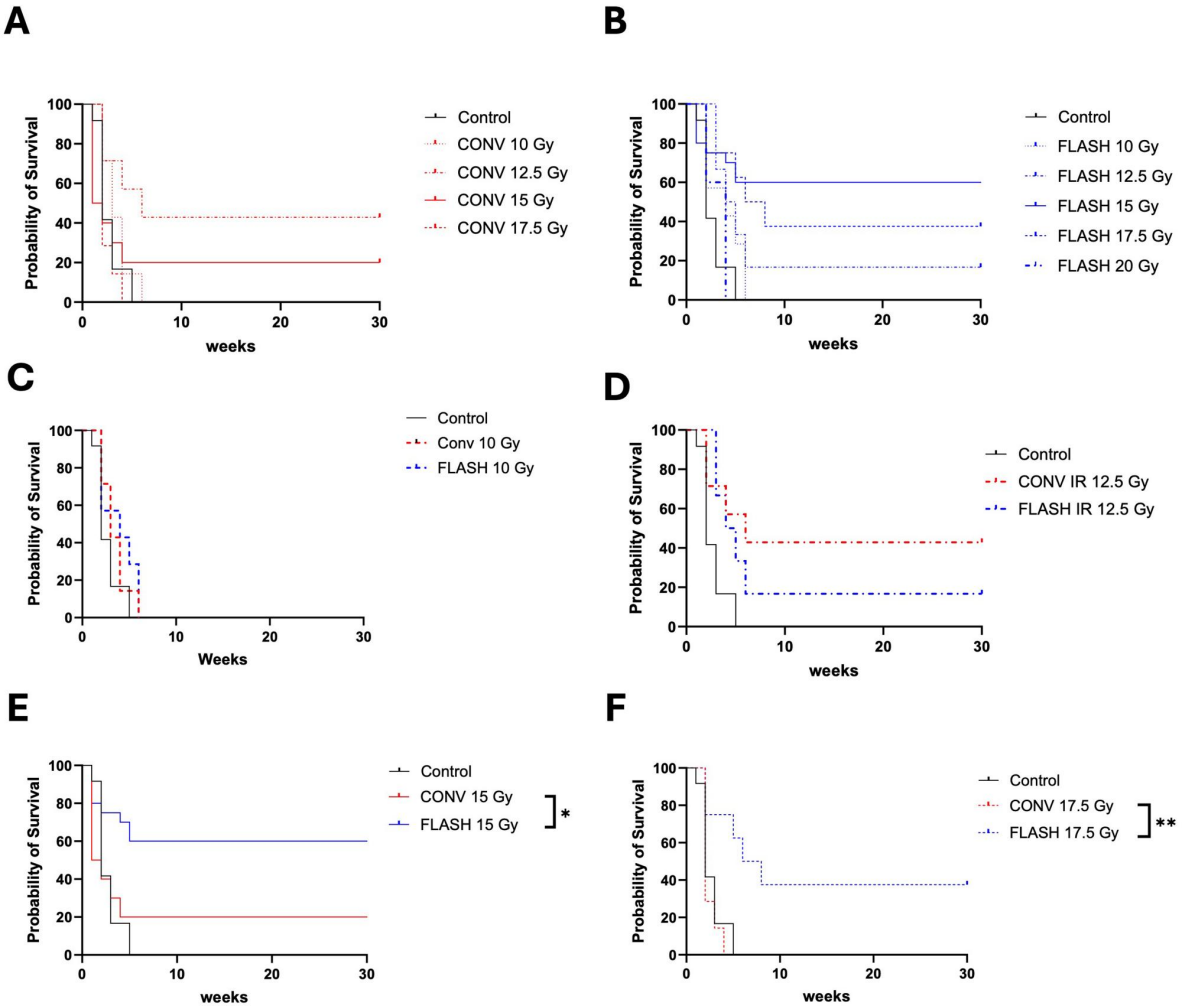
FLASH RT improves survival in orthotopic MBT2 bladder tumor models compared to CONV RT at higher doses. Kaplan-Meier survival curves for orthotopic MBT2 tumors in C3H mice treated with CONV (A) or FLASH (B) RT across escalating doses. Significant survival differences could be found for 10 (C), 12.5 Gy (D), 15 Gy (E, including both dose rates), and 17.5 Gy treatments (F).*P < .05, **P < .01.

Table 1 and Figure 3 summarize the survival outcomes for the orthotopic MIBC model following CONV and FLASH radiotherapy across a range of doses. The control group had a median survival of 2 weeks. Both 10 and 12.5 Gy doses modestly extended survival, with CONV RT resulting in median survivals of 3 and 6 weeks, respectively, while FLASH RT achieved 4 and 4.5 weeks. At these lower doses, there were no significant differences between FLASH and CONV (*P = .*5 for both comparisons). Long-term survival beyond the 30-week endpoint was rare at these doses, with only 1 of 6 FLASH-treated and 3 of 7 CONV-treated mice surviving after 12.5 Gy.

**Table 1:** Median survival (weeks) for orthotopic C3H MIBC model.

Control	2 (12)				
	10 Gy	12.5 Gy	15 Gy	17.5 Gy	20 Gy
CONV radiotherapy	3 (7)	6 (7)	1.5 (10)	2 (7)	
FLASH radiotherapy	4 (7)	4.5 (6)	>30 (20)^a^	7 (8)	4 (5)

The number in parentheses is the number of mice in each group.

a Including *n = *10 for 10^6^ Gy/s and *n = *10 for 10^2^ Gy/s averaged dose rates.

A dramatic difference emerged at 15 Gy, where FLASH RT resulted in a median survival greater than 30 weeks, with 12 of 20 mice (60% for both ∼10^6^ Gy/s and 10^2^ Gy/s dose rate) still alive at the study endpoint. This was significantly longer than the CONV 15 Gy group, which had a median survival of only 1.5 weeks and just 2 of 10 mice (20%) surviving to the endpoint (*P = .*02). Similarly, at 17.5 Gy, FLASH treatment extended median survival to 7 weeks compared to 2 weeks for CONV, with 3 of 7 mice (43%) in the FLASH group still alive at 30 weeks (*P = .*004). Interestingly, at 20 Gy, FLASH RT did not confer further benefit, with median survival dropping to 4 weeks and no mice surviving to the endpoint, suggesting possible toxicity or diminishing returns at this dose.

No significant difference in tumor growth was seen between FLASH and CONV RT (Figure S5). Notably, toxicity-related euthanasia, defined as >15% from pre-treatment weight or predefined clinical signs of treatment-related distress in the absence of tumor progression (>450 mm³), was observed across all groups (Table 2), including 16.7% of control mice. In the CONV treated groups, toxicity-related euthanasia increased with dose: 28.6% following 10 Gy treatment, 42.9% for 12.5 Gy, 50% for 15 Gy, and 71.4% for 17.5 Gy. On the other hand, toxicity-related and dose dependent euthanasia was also observed in the FLASH treated groups: with 14.3% following 10 Gy treatment, 16.7% for 12.5 Gy treatment, 30% for 15 Gy, 62.5% for 17.5 Gy, and 60% for 20 Gy. These findings indicate that FLASH and CONV RT result in similar tumor response at matched doses. Severe hematuria was observed more frequently in CONV-treated mice, particularly at higher doses, and was generally less common in FLASH-treated animals. The reduced rate of toxicity-related euthanasia in FLASH-treated mice compared to those receiving CONV RT suggests lower treatment-associated toxicity and improved overall survival.

**Table 2:** Toxicity related euthanasia *g* for orthotopic C3H MIBC model.

Control	16.7% (2/12)				
	10 Gy	12.5 Gy	15 Gy	17.5 Gy	20 Gy
CONV radiotherapy	28.6% (2/7)	42.9% (3/7)	50% (5/10)	71.4% (5/7)	
FLASH radiotherapy	14.3% (1/7)	16.7% (1/6)	30% (6/20)	62.5% (5/8)	60% (3/5)

Values are presented as percentages, with the number of mice euthanized due to toxicity shown in parentheses as *x*/*n*, where *x* is the number of affected mice and *n* is the total number of mice in the group.

### Effect of dose rate and fractionation

**Figure 4:**
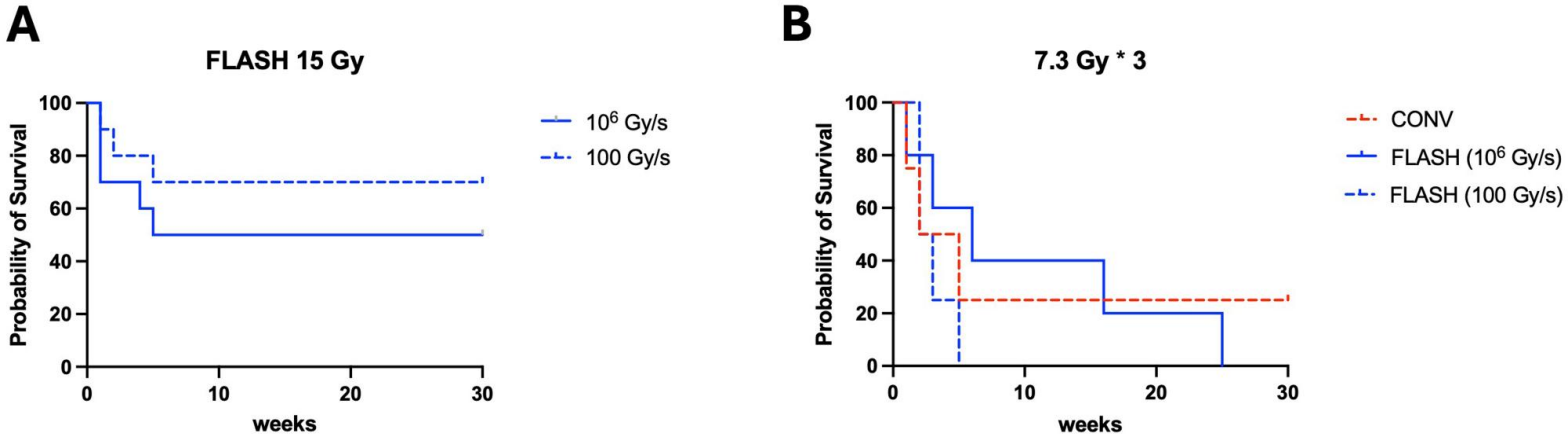
Effect of dose rate and fractionation on survival following 15 Gy RT in orthotopic MBT2 tumor-bearing mice. (A) Comparison of survival outcomes after FLASH RT administered at varying dose rates. No significant difference in survival was observed, though a trend toward improved outcomes at 10^6^ Gy/s was noted. *n = *10 for each group. (B) Assessment of survival following CONV and FLASH RT delivered at hypofractionated schedule (7.3 Gy × 3 fractions, daily). Differences were not statistically significant, but a trend favoring FLASH at higher dose rate was observed. *n = *4 for CONV and FLASH (10^2^ Gy/s) and *n = *5 for FLASH (10^6^ Gy/s).

Two different dose rates, ∼10^6^ Gy/s and 10^2^ Gy/s, were evaluated for 15 Gy FLASH RT treatment, but no significant difference in survival was observed (Figure 4A; 10^6^ Gy/s vs. 10^2^ Gy/s: *P = .*4). Additionally, FLASH and CONV treatment were also delivered in a (hypo-)fractionated setting, using 7.3 Gy delivered over 3 consecutive daily fractions, biologically equivalent (dose, BED: 37.9 Gy vs. 37.5 Gy) to a single 15 Gy fraction, assuming an α/β of 10 (Figure 4B). While no statistically significant differences were found among the groups, there was a trend suggesting worse survival outcomes for FLASH at 10^2^ Gy/s compared to FLASH at 10^6^ Gy/s (CONV vs. FLASH 10^6^ Gy/s: *P = .*9; CONV vs. FLASH 10^2^ Gy/s: *P = .*6; FLASH 10^6^ Gy/s vs. FLASH 10^2^ Gy/s: *P = .*1). Among the fractionated groups, median survival was longest in the FLASH 7.3 Gy × 3 (10^6^ Gy/s) group (6 weeks). In contrast, CONV 7.3 Gy × 3 resulted in a median survival of 3.5 weeks, whereas FLASH 7.3 Gy × 3 (10^2^ Gy/s) had the shortest median survival of 2.5 weeks. The tumor growth curve demonstrated a similar reduction in growth for both dose rates at 15 Gy or fractionation at both modalities (Figure S6A-C).

### Acute systemic toxicity

**Figure 5:**
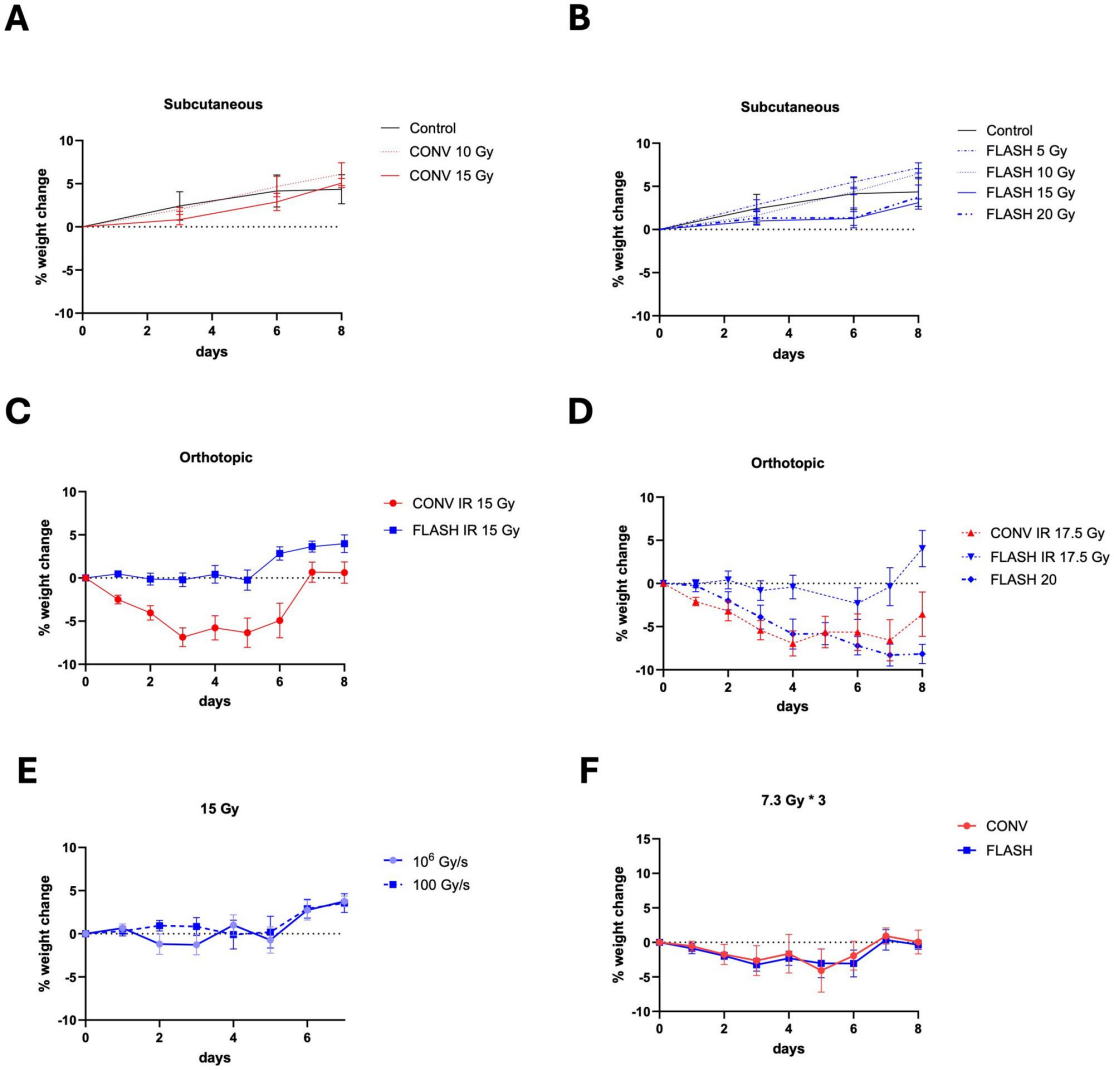
Assessment of acute systemic toxicity based on post-treatment weight changes in mice receiving CONV or FLASH RT. (A and B) Subcutaneous MBT2 tumor models showed minimal acute weight loss across all treatment groups, with no significant differences between FLASH, CONV, or control mice within 8 days post-RT. (C and D) In orthotopic tumor models, significant weight differences were observed between FLASH and CONV RT at both 15 Gy and 17.5 Gy, indicating greater systemic toxicity with CONV RT. (E and F) No significant differences in weight loss were observed following FLASH RT with varying dose rates (10^6^ vs. 10^2^ Gy/s) or with fractionated regimens (7.3 Gy × 3), suggesting consistent systemic tolerance across FLASH delivery conditions.

To evaluate systemic toxicity, post-RT body weight was monitored as a surrogate marker for acute treatment-related adverse effects. In the subcutaneous tumor model, all treatment groups, including control, CONV, and FLASH RT at varying doses, exhibited minimal acute weight loss. No significant weight differences were detected between groups within the first 8 days post-treatment, indicating low systemic burden and good tolerability of both modalities in this model (Figure 5A and B).

In contrast, the orthotopic tumor model revealed a distinct pattern. At the radiation doses of 15 and 17.5 Gy, mice receiving CONV RT experienced significantly greater weight loss compared to those treated with FLASH RT, suggesting increased systemic toxicity associated with conventional dose delivery (Figure 5C and D; *P < .*0001). When the FLASH RT dose was escalated to 20 Gy, the resulting weight loss mirrored that observed in the 17.5 Gy CONV RT group, suggesting that at very high doses, the protective effect of FLASH RT is not enough to limit toxicity.

Further analyses explored whether variations in FLASH RT delivery, either through altered dose rates (10^6^ vs. 10^2^ Gy/s) or fractionation (7.3 Gy × 3), would influence systemic toxicity. However, no notable differences in weight change were observed among these groups (Figure 5E and F), suggesting that neither dose rate modulation nor fractionated schedules significantly impacted acute systemic side effects under the conditions tested.

### Late normal tissue toxicity

Representative Masson’s trichrome staining of colon tissue collected 30 weeks post-irradiation (Figure S7A) demonstrated qualitatively reduced collagen deposition following FLASH RT compared with CONV-treated mice. Quantitative image analysis of collagen-positive area in the colon showed an overall decrease after FLASH, with mean values of approximately 28% for CONV 15 Gy (*n = *2), 22% for CONV 7.3 Gy × 3 (*n = *1), and 15-20% for FLASH-treated groups (FLASH 15 Gy ≈ 15% [*n = *4], FLASH 15 Gy (10^2^ Gy s^−1^) ≈ 20% [*n = *7], FLASH 17.5 Gy ≈ 16%[*n = *4]) (Figure S7B). Limited histological analysis of bladder tissue from the same experimental cohorts showed a comparable trend (Figure S7B). Collagen deposition in the bladder wall was lower after FLASH compared with CONV irradiation (mean collagen area: CONV 12.5 Gy = 76.5% [*n = *1]; CONV 7.3 Gy × 3 = 79.6% [*n = *1]; FLASH 15 Gy (10^2^ Gy s^−1^) = 30.4% [*n = *1]; FLASH 15 Gy = 52.9 ± 7.0% [*n = *5]; FLASH 17.5 Gy = 51.8 ± 5.3% [*n = *3]). Given the small number of evaluable specimens, these data are presented descriptively and not subjected to statistical analysis. Nevertheless, the consistent reduction in collagen deposition across both organs supports the interpretation of lower late tissue fibrosis and enhanced normal tissue preservation with FLASH RT.

### Tumor microenvironmental responses

Multiplex immunofluorescence staining for SPP1, F4/80, vimentin and DAPI was performed on orthotopic and subcutaneous tumor sections within 1 to 2 weeks’ post-irradiation to assess ECM remodeling and inflammatory cell infiltration following irradiation (Figure S8). As MBT2 tumor cells are intrinsically vimentin-positive, vimentin staining served as a tumor-cell marker. FLASH-treated tumors exhibited higher SPP1 signal, consistent with the ECM-remodeling and stress-adaptive transcriptional programmes identified in the RNA-seq analysis. In contrast, CONV-treated tumors displayed increased F4/80^+^ macrophage infiltration, indicating greater inflammatory recruitment and tissue irritation. These modality-specific patterns in SPP1 and F4/80, though non-quantitative, support the broader trend of reduced inflammatory and fibrotic remodeling following FLASH irradiation.

## Discussion

FLASH RT has been shown to significantly reduce normal tissue toxicity while maintaining tumor control comparable to that of CONV RT in multiple studies.^13^^,^^16^^,^^18^^,^^29^ Here, we evaluate its effects *in vitro* and *in vivo* using MIBC models. *In vitro*, clonogenic assay revealed equivalent survival between the 2 modalities (Figure S2). Although clonogenic survival did not differ significantly between CONV and FLASH irradiation, the fitted survival curves yielded different numerical α/β ratios. In principle, a higher α/β ratio can indicate a greater contribution of irreparable, directly lethal lesions, whereas a lower α/β ratio may suggest a shift toward sublethal or repairable forms of damage—mechanistic patterns that have been proposed in the context of UHDR irradiation and radical chemistry modulation.^30–33^ However, in our dataset these α/β differences should be interpreted with caution. For both modalities, the β parameters had wide confidence intervals that crossed zero, indicating that the curvature of the dose-response relationship was poorly constrained and that the survival decline is also compatible with an approximately exponential form. Consequently, the α/β ratios are not statistically reliable indicators of mechanistic differences. The near-superimposable survival curves and overlapping confidence intervals for α and β support the conclusion that MBT2 cells exhibit no meaningful difference in radiosensitivity between CONV and FLASH irradiation *in vitro*, and mechanistic distinctions between the modalities should not be inferred from these fitted parameters alone. In addition, it is important to consider that clonogenic survival assays may be influenced by cell-cell cooperation effects, particularly at higher seeding densities, whereby irradiated cells can support each other’s survival and colony formation through paracrine signaling or shared microenvironmental factors.^34^ Such cooperative interactions can reduce apparent curvature and complicate interpretation of linear-quadratic fitting parameters. While our seeding strategy was optimized to remain within standard assay conditions, we cannot fully exclude the possibility that subtle cooperation effects contributed to the poorly constrained β estimates. Nevertheless, because both FLASH and CONV irradiations were performed under identical experimental conditions and yielded near-superimposable survival curves with overlapping confidence intervals, any potential cooperation effect would be expected to influence both modalities similarly and therefore does not alter our conclusion that intrinsic radiosensitivity was comparable between FLASH and CONV *in vitro*.

Although no difference in clonogenic survival was observed under standard culture conditions, it is well established that oxygen tension strongly influences cellular radiosensitivity and may modulate the magnitude of the FLASH effect. Most *in vitro* studies, including ours, are conducted under normoxic conditions (21% O_2_), whereas *in vivo* tissues experience substantially lower oxygen levels, typically 2%-8% O_2_ in normal tissues and <2% O_2_ in hypoxic tumor regions.^35^ Several *in vitro* studies have shown that the appearance and magnitude of a FLASH sparing effect can depend strongly on oxygen tension: for instance, DU145 cells showed no sparing in normoxia but clear sparing at ∼1.6% O_2_ (for doses >15 Gy),^36^ A549/H1437 cultures exhibited FLASH sparing only under low O_2_ (≈1%) and at doses >8 Gy,^37^ and H454 cells had a larger UHDR-associated survival increase at 4% O_2_ than in normoxia.^38^ These observations indicate that oxygenation is a critical experimental parameter when interpreting *in vitro* UHDR results.^39^^,^^40^ Such oxygen-dependent effects may therefore contribute to the comparable clonogenic outcomes observed here under normoxia.

Gene expression analysis, nevertheless, revealed marked transcriptional divergence at 24-hour post 10 Gy IR. Suggesting that even when survival is similar, the cellular responses differ substantially at the molecular level (Figure 1A). This divergence likely reflects early transcriptional and stress-response programmes that do not immediately translate into macroscopic differences in clonogenic survival or tumor growth. Many of the genes modulated by FLASH are involved in cellular adaptation and tissue remodeling rather than proliferation.

GO analysis uncovered distinct transcriptional responses (Figure 1B). While both modalities induced widespread transcriptional changes, CONV IR was associated with reduced expression in pathways related to ribosome biogenesis, ER stress response, ECM organization, and immune function. FLASH IR, in contrast, appeared to trigger activation in several stress- and metabolism-related pathways, potentially reflecting a distinct mode of cellular response (Figure 1C). Notably, FLASH upregulated *Spp1* (osteopontin), *Vim* (vimentin) and *Lgals1* (galectin-1), while downregulating *Hspa5* (BiP/GRP78), *P4hb*, *Rpl30* and *Actg1*, genes associated with protein folding, translation and cytoskeletal organization. The upregulation of translation-associated genes alongside downregulation of ribosomal subunits may suggest a regulatory shift, but further mechanistic studies would be needed to confirm this. Stress response genes upregulate redox regulators and cytoskeletal elements, while ER stress chaperones are suppressed. Glycolytic genes are variably regulated, suggesting altered metabolism. FLASH IR promotes genes for ECM remodeling and migration but lowers those for cytoskeletal stability. Immunologically, inflammatory mediators increase, while antigen presentation via MHC class I decreases. These transcriptional changes raise hypotheses regarding altered redox regulation, metabolism, and immune modulation following FLASH IR, though the *in vitro* nature of the experiment limits interpretation. Importantly, these findings should be viewed as exploratory, and further validation using *in vivo* tissues that preserve tumor-host and immune interactions will be essential. While full transcriptomic profiling of orthotopic tumors was beyond the scope of this study, multiplex immunofluorescence staining for SPP1 and F4/80 provided *in vivo* support for the RNA-seq-derived pathway differences, with FLASH tumors exhibiting increased SPP1 expression and CONV tumors showing greater macrophage infiltration (Figure S8). Notably, the increase in SPP1 occurred at early time points (1-2 weeks post-irradiation) and did not translate into greater late-stage fibrosis; instead, late collagen deposition was lower after FLASH (Figure S7), suggesting that early SPP1 induction reflects adaptive, transient ECM remodeling rather than chronic fibrotic progression.

To explore physiological aspects of the FLASH effect in normal tissues, we additionally examined intestinal organoids as a representative healthy-epithelial model. Despite receiving equivalent radiation doses, FLASH-irradiated organoids maintained significantly greater size and normal morphology compared with those irradiated at CONV dose rates (*P < .*001), indicating enhanced tissue tolerance and regenerative capacity. This differential response supports a physiological contribution to the FLASH effect beyond radiochemical mechanisms and parallels the normal tissue sparing and reduced fibrosis observed *in vivo*. The observed FLASH-sparing effect in intestinal organoids, despite the relatively low irradiation doses (2.5-5 Gy), may in part reflect the microenvironmental characteristics of 3D organoid culture. Organoids grown within Matrigel domes develop diffusion-limited oxygen gradients, with oxygen tensions likely reduced to physioxic or mildly hypoxic levels (≈1%-5% O_2_) within the matrix.^41^^,^^42^ Such conditions could enhance sensitivity to ultra-high dose rate effects through transient oxygen depletion and altered radical chemistry. This may explain why a measurable protective effect was observed in organoids under conditions where the FLASH effect is typically less pronounced in fully oxygenated 2D cultures.

FLASH and CONV RT elicited equivalent tumor growth delays in subcutaneous MIBC models at 10 and 15 Gy, with median tripling times of 2.5-3 weeks versus 1.5 weeks in untreated controls. This lack of differentiation in the subcutaneous model aligns with previous studies, showing similar tumor response (mainly tumor growth delay) following FLASH and CONV RT.^43^ In contrast, the orthotopic MIBC models revealed a substantial benefit from FLASH RT: 15 Gy yielded >30 weeks median survival versus 1.5 weeks with CONV RT (*P = .*02, Table 1). FLASH thus enabled safe dose escalation, achieving 60% long-term survival at 15 Gy compared to 20% for CONV.

Both FLASH and CONV RT lose efficacy at very high single doses (17.5-20 Gy), with diminished long‐term survival and increased toxicity‐related euthanasia, indicating a toxicity ceiling beyond which acute effects prevail over FLASH sparing. Notably, FLASH groups exhibited lower toxicity‐related euthanasia rate than CONV at matched doses (e.g., 30% vs. 50% at 15 Gy), pointing to normal‐tissue protection. These observations mirror preclinical studies in other organs where FLASH RT reduces intestinal crypt loss and weight‐loss toxicity without compromising tumor kill.^12^^,^^15^ This finding underscores the importance of optimizing dose regimens to balance tumor control and toxicity. Histological examination of both the colon and bladder, supported by limited quantitative assessment, indicated reduced collagen deposition following FLASH RT compared with CONV RT (Figure S7), consistent with a protective effect on normal tissues. Although the number of evaluable specimens was small, these observations collectively support the interpretation that ultra-high dose rate irradiation mitigates late normal-tissue fibrosis across multiple organs, aligning with previous reports of reduced late-effect toxicity at UHDR dose rates.^13^^,^^44^^,^^45^

We found no significant survival differences between FLASH delivered at 10^2^ Gy/s vs. 10^6^ Gy/s (*P = .*4), consistent with data showing that above a threshold (∼10^2^ Gy/s), the FLASH effect plateaus.^46^ Conversely, fractionating FLASH into three 7.3 Gy doses abolished its advantage, reducing median survival to 2.5 weeks vs. 3.5 weeks for CONV, aligning with reports that per-fraction dose is critical for maintaining normal tissue sparing.^47–49^ This dose-fraction interplay suggests that very high (≥10 Gy) fraction doses may be required to retain FLASH advantages in the clinic, and/or that even higher dose rates are required for a maintained advantage at lower fraction doses. The subtle differences observed between 10^2^ Gy/s and 10^6^ Gy/s FLASH deliveries may reflect the importance of instantaneous dose rate and dose per-pulse for maintaining normal-tissue sparing, particularly at lower per-fraction doses. A high instantaneous/average dose rate (in the 10^6^ Gy/s range) delivered in single pulses has been reported to be necessary to sustain the FLASH effect under such conditions.^50^^,^^51^ Similar tissue-specific dependencies have been described, where intestine required higher dose-per-pulse or instantaneous dose rate thresholds than skin to exhibit sparing.^15^^,^^52^ The apparent anti-FLASH effect observed at 10^2^ Gy/s is likely within the biological variability of the model rather than indicative of a true reversal of the effect. Collectively, these observations suggest that when per-fraction doses are small, achieving a sufficiently high instantaneous dose rate is critical for preserving the FLASH advantage, consistent with prior intestinal and brain studies.

The equivalent tumor control in subcutaneous models supports the broad antitumor applicability of FLASH RT. Our orthotopic findings highlight its unique value for dose escalation against high-risk tumors like MIBC. Yet the toxicity ceiling at very high doses cautions against indiscriminate escalation. We acknowledge that the use of only female mice represents a limitation of the current study in terms of clinical translation, although this choice was dictated by technical considerations inherent to the orthotopic bladder model. However, the underlying physical and radiochemical mechanisms governing the FLASH effect are not expected to differ between sexes, and future studies including both male and female animals will be important to confirm the generalizability of these findings. Moreover, the restricted penetration depth of electron-based FLASH presents intrinsic limitations that may affect clinical translatability, limiting its utility for deep-seated tumors like MIBC, and the lack of widespread clinical infrastructure for delivering uniform electron FLASH complicates broader adoption.

From a clinical perspective, single-dose FLASH regimens may be challenging to implement for bladder cancer, particularly in adjuvant or bladder-preserving settings where fractionated schedules are standard. Therefore, further preclinical optimization of fractionated or hypofractionated FLASH protocols is warranted to identify dose-per-fraction and instantaneous/average dose rate thresholds that maintain normal-tissue sparing during repeated exposures. Studies using advanced normal-tissue models, such as bladder and intestinal organoids, together with in-vivo validation, will be instrumental in defining clinically feasible parameters and informing the design of future bladder-preserving FLASH radiotherapy approaches. On the other hand, first-in-human trials (FAST-01 and FAST-02 for bone metastases) using proton FLASH demonstrate feasibility and safety, paving the way for further bladder cancer studies.^19^^,^^20^ Given that trimodal therapy (TMT) remains the standard of care for MIBC, it is unlikely for FLASH RT to serve as a standalone therapy in the near term. Instead, its potential may lie in enhancing TMT, for instance by enabling higher biologically effective doses with reduced toxicity, and potentially augmenting the immunogenic effects of radiation, as explored in the KEYNOTE-922 and IMMUNOPRESERVE trials.^53^^,^^54^ Future research will focus on dissecting the biological mechanisms, refining treatment strategies, and evaluating long-term outcomes to facilitate the clinical translation of FLASH RT. Collectively, our data and the broader literature suggest FLASH RT holds significant promise for optimized, organ-sparing dose escalation in MIBC, if dosing regimens are carefully tailored to balance tumor control and treatment tolerance.

## Supplementary Material

tqag071_Supplementary_Data
