# Supplementary material for: FLASH radiotherapy enables dose escalation resulting in improved survival in an orthotopic muscle-invasive bladder cancer mouse model
Source: Br J Radiol. 2026 Mar 26;99(1182):1101–13. doi: 10.1093/bjr/tqag071 (PMC13273415; doi:10.1093/bjr/tqag071)
Supplement: tqag071_Supplementary_Data [file tqag071_supplementary_data.zip › 20260227_Supplementary information_clean.docx]

**Supplementary information**

*Methods*

IR setup

Cells were irradiated in T12.5 flasks, positioned upright (bottom of flask facing the beam) just downstream of a 6 mm brass collimator with a 50 mm in diameter central aperture, with the flask centred in the aperture[33]. The mice were anesthetised using isoflurane supplemented with (1 part) air mixed with (1 part) 95% oxygen (4% for anaesthetic induction and 2% for maintenance, with total anaesthesia time <10 minutes). Once induced mice were individually placed in a mouse cradle[34] in front of the horizontal radiation beam.

Organoids were irradiated *in situ* within Matrigel domes using the same 6 MeV electron beamline employed for cell and tumour irradiation. CONV irradiation was delivered at ~0.1 Gy s⁻¹, whereas FLASH irradiation was delivered at ≥2,000 Gy s⁻¹ using 3.4 µs electron pulses at 300 Hz. Organoid domes were exposed to single fractions of 2.5 Gy or 5 Gy (CONV or FLASH). Dosimetry and pulse structure were identical to those described for *in vitro* MBT2 cell irradiation.

Dose fractionation parameter

For the orthotopic tumour treatments, we also tested the average FLASH dose rate dependence by delivering the 15 Gy FLASH treatment in a single 3.4 µs electron pulse (dose rate = 4.5 x 10^6^ Gy/s) or in multiple pulses (at 300 Hz) for an average dose rate of ≈10^2^ Gy/s, as well as the effect of fractionation by delivering the treatment in 7.3 Gy x 3 fractions, over 3 consecutive days, with dose rates of 0.1 Gy/s, 10^2^ Gy/s, and a single 3.4 µs electron pulse (dose rate = 2.2 x 10^6^ Gy/s).

Dosimetry

GafChromic EBT-XD film (Ashland Inc, Covington, KY) was used to verify the prescribed dose before and after mice treatments. The film was positioned at the surface of a mouse (Perspex) phantom positioned as per the mice in the beam path[15]. Films were scanned (Epson Perfection v850 Pro, Seiko Epson Corporation, Nagano, Japan) 24h post-irradiation (IR) and the red channel analysed with ImageJ (version 1.52a, Wayne Rasband, National Institutes of Health). The measured film dose was averaged over a 20x20 (*in vitro*), 7x7 (subcutaneous tumour) or 9x12 (orthotopic tumour) mm^2^ central area of the exposed part of the. The films had previously been calibrated in a 6 MeV clinical electron beam from a Varian Truebeam (Varian Medical Systems Inc, Palo Alto, CA) linear accelerator, at the Churchill Hospital site in Oxford, UK. For online verification of the dose delivery, an Advanced Markus (PTW-Freiburg GmbH, Freiburg, Germany) ionisation chamber was used as a beam monitor (corrected for reduced ion collection efficiency in ultra-high dose rate beams[35]), as well as a beam energy monitor to verify that the electron beam energy was consistently 6 MeV[19]. Our overall uncertainty in dosimetry was estimated to be 4% (including a measured output variation of our FLASH and CONV beam deliveries of within 2%).

Clonogenic assay

MBT2 cells were seeded onto T12.5 flasks (Falcon) at 50,000 cells per flask using 0.25% trypsin-EDTA (with phenol red, Gibco) 18 hours before IR. The flasks were irradiated with 5, 10, 15 or 20 Gy at FLASH and CONV dose rates. After IR, cells were trypsinised and re-seeded to set up the clonogenic assay at appropriate seeding density on 10 cm dishes. Cells were incubated for two weeks to allow colony formation. Colonies were then fixed with methanol, stained with 0.01% (w/v) crystal violet, and manually counted. Only colonies containing 50 or more cells were considered viable for counting. Surviving fractions were calculated by normalising colony counts to the plating efficiency of non-irradiated control samples.

Clonogenic survival data were fitted using a weighted linear-quadratic (LQ) model,

$S(D)=\exp(-\alpha D-\beta D^{2})$, implemented in GraphPad Prism. Weights were proportional to the inverse variance of each data point. Fits were unconstrained, allowing $\beta$ to take positive or negative values. Parameter confidence intervals were derived using non-linear least-squares regression with 10,000 bootstrap iterations. Model adequacy was assessed using R² values and residual analysis.

Bulk RNA isolation and sequencing analysis

MBT2 cells were seeded onto T12.5 flasks (Falcon) at 50,000 cells per flask and incubated overnight with 0.25% trypsin-EDTA (with phenol red, Gibco) before irradiation (IR). Flasks were exposed to 0, 5, or 10 Gy at either FLASH or CONV dose rates. After 24 hours, cells were harvested, snap-frozen in liquid nitrogen, and stored at -80 °C until processing. For each condition, RNA extracted from three independent biological replicates was pooled prior to sequencing to obtain sufficient material and reduce sample-to-sample variability.

RNA extraction and bulk RNA sequencing were performed by Eurofins Genomics EU (Konstanz, Germany) using their standard transcriptome sequencing service. Poly(A)-enriched RNA libraries were prepared and sequenced on an Illumina NovaSeq platform, generating 150 bp paired-end reads with an average depth of ~40 million reads per sample. Quality control was performed using FastQC, and adaptor and quality trimming were conducted with Trimmomatic. The cleaned reads were aligned to the mouse reference genome (GRCm38/mm10, Ensembl v90) using Bowtie, and splice junctions were identified with TopHat. Transcript assembly and quantification were carried out using Cufflinks, and transcript models from individual samples were merged using Cuffmerge. Differential expression analysis was performed at both the transcript and gene level using Cuffdiff , and log₂ fold-change values were used for ranking genes between conditions. Gene set enrichment analysis (GSEA) was then performed on these ranked lists to identify pathways enriched under FLASH or CONV irradiation. Pathway significance was determined based on the GSEA-derived false discovery rate (FDR), calculated through permutation testing (independent of biological replicates). Enrichment results are reported as normalized enrichment score (NES) values with corresponding FDRs.

Gene Ontology (GO) enrichment analysis of differentially expressed genes (DEGs) was performed using the g:Profiler web server (https://biit.cs.ut.ee/gprofiler), using the *Mus musculus* (Ensembl v90) reference. The analysis focused on the Biological Process category and included DEGs with a false discovery rate (FDR) < 0.05 and |log₂ fold-change| > 1. The background gene set was automatically defined by g:Profiler based on the organism-wide gene universe. Enrichment was assessed using a hypergeometric test with Benjamini–Hochberg false discovery rate correction, and only terms with adjusted p-values < 0.05 were considered significantly enriched.

To visualise transcriptional differences among conditions, a focused heatmap was generated using selected genes representing key pathways identified by the RNA-seq analysis. Normalised expression values (FPKM) were log₂-transformed and row-wise z-scored. Hierarchical clustering was performed using correlation distance (1 – Pearson correlation) and Ward linkage, implemented in SciPy, to better reflect global transcriptomic relationships observed in PCA. Heatmaps and dendrograms were generated using Seaborn’s clustermap function.

Histology and fibrosis quantification
Colon and bladder tissues were collected at 30 weeks post-irradiation from mice that survived to the study endpoint. Tissues were fixed in 10% neutral-buffered formalin for 24 hours, processed, paraffin-embedded, and sectioned at 4 µm. Masson’s trichrome staining (Cat#RBK-0601-00B from Cell Path for colon, or HT15 from Sigma-Alderich for bladder) was performed according to the manufacturer’s protocol to visualise collagen deposition (green for colon and blue for bladder) within the tissue.

Stained slides were digitised using a bright-field slide scanner (Zeiss Axioscan 7) under identical acquisition settings. Collagen-positive area was quantified using ImageJ/Fiji by colour deconvolution followed by thresholding of the collagen channel. Regions of interest were drawn to include the full thickness of the colon or bladder. Collagen area was expressed as a percentage of total tissue area within the region of interest.

Multiplex immunofluorescence staining.
Orthotopic tumour tissues were fixed in 10% neutral-buffered formalin for 24 hours, paraffin-embedded, and sectioned at 5 µm. Multiplex immunofluorescence staining was performed on FFPE sections using fluorophore-conjugated primary antibodies. The staining panel consisted of SPP1 (Alexa 488, extracellular matrix remodelling), F4/80 (Alexa 647, macrophages), vimentin (Alexa 555, tumour-cell marker), and DAPI (nuclear counterstain). Heat-induced antigen retrieval (citrate buffer, pH 6.0 and Tris-EDTA buffer, pH 9.0) was performed prior to blocking in 5% normal serum.

Stained slides were mounted in antifade medium and imaged using a fluorescence scanner (CellDIVE, Leica) under identical exposure and gain settings across samples to allow qualitative comparison.

**Supplementary Figures**

**
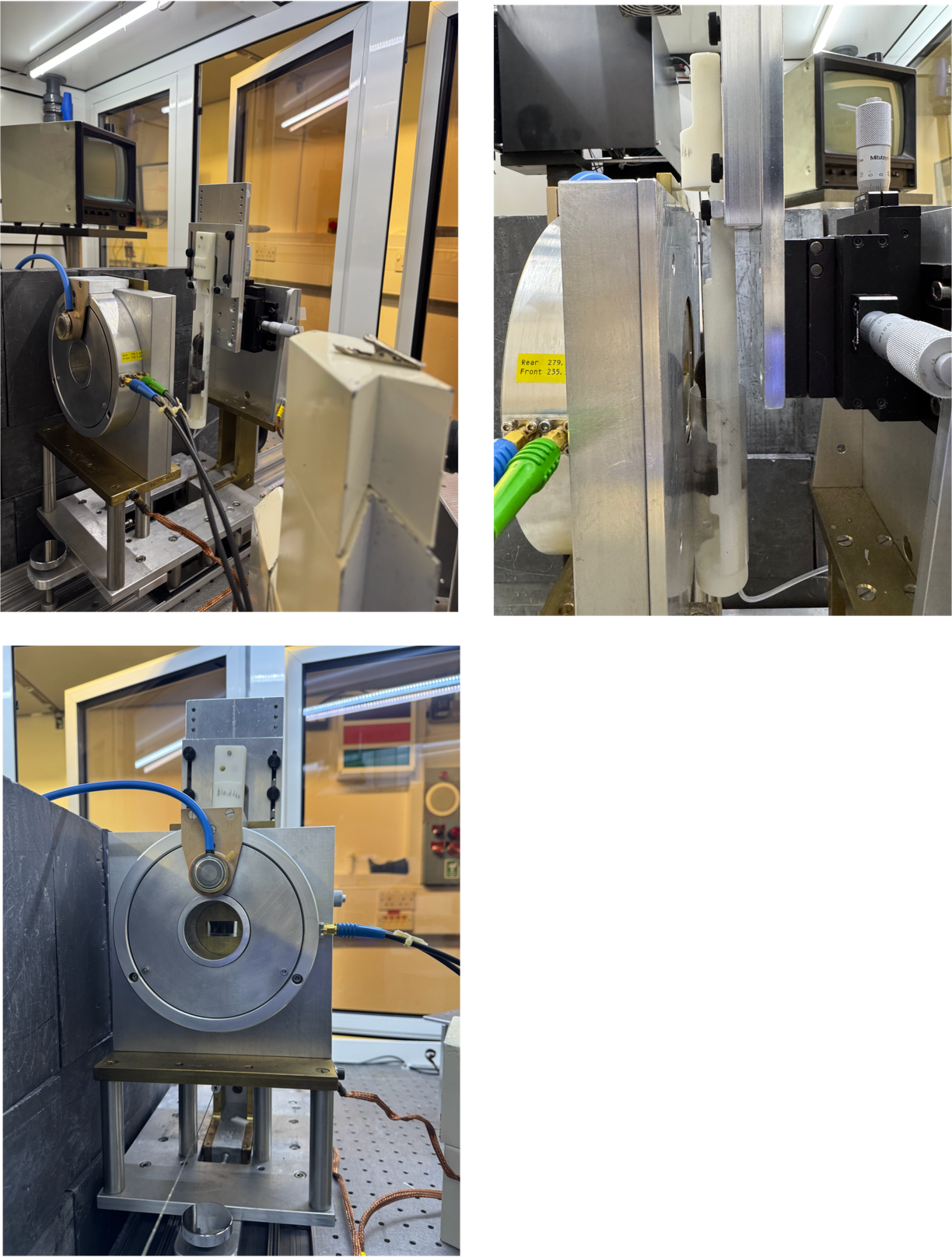
**

**Figure S1. Schematic illustration of the irradiation setup used for orthotopic MBT2 bladder tumours.** Photographs show the experimental arrangement used during treatment, including the inverted mouse positioning, which displaces the intestines cranially to minimise gastrointestinal irradiation, and the 6-mm brass collimator fitted with a 15 × 30 mm² rectangular aperture used to confine the beam to the lower abdomen and target the bladder region.


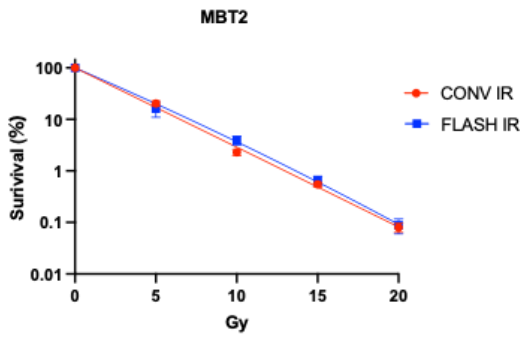


**Figure S2.** Clonogenic survival curves of MBT2 cells exposed to conventional dose rate (CONV, ≈ 0.1 Gy s⁻¹) or ultra-high dose rate (FLASH, ≥ 2,000 Gy s⁻¹) electron irradiation. Data points represent mean ± SEM (n = 4–5 independent experiments per condition); curves were fitted using a weighted linear-quadratic model.


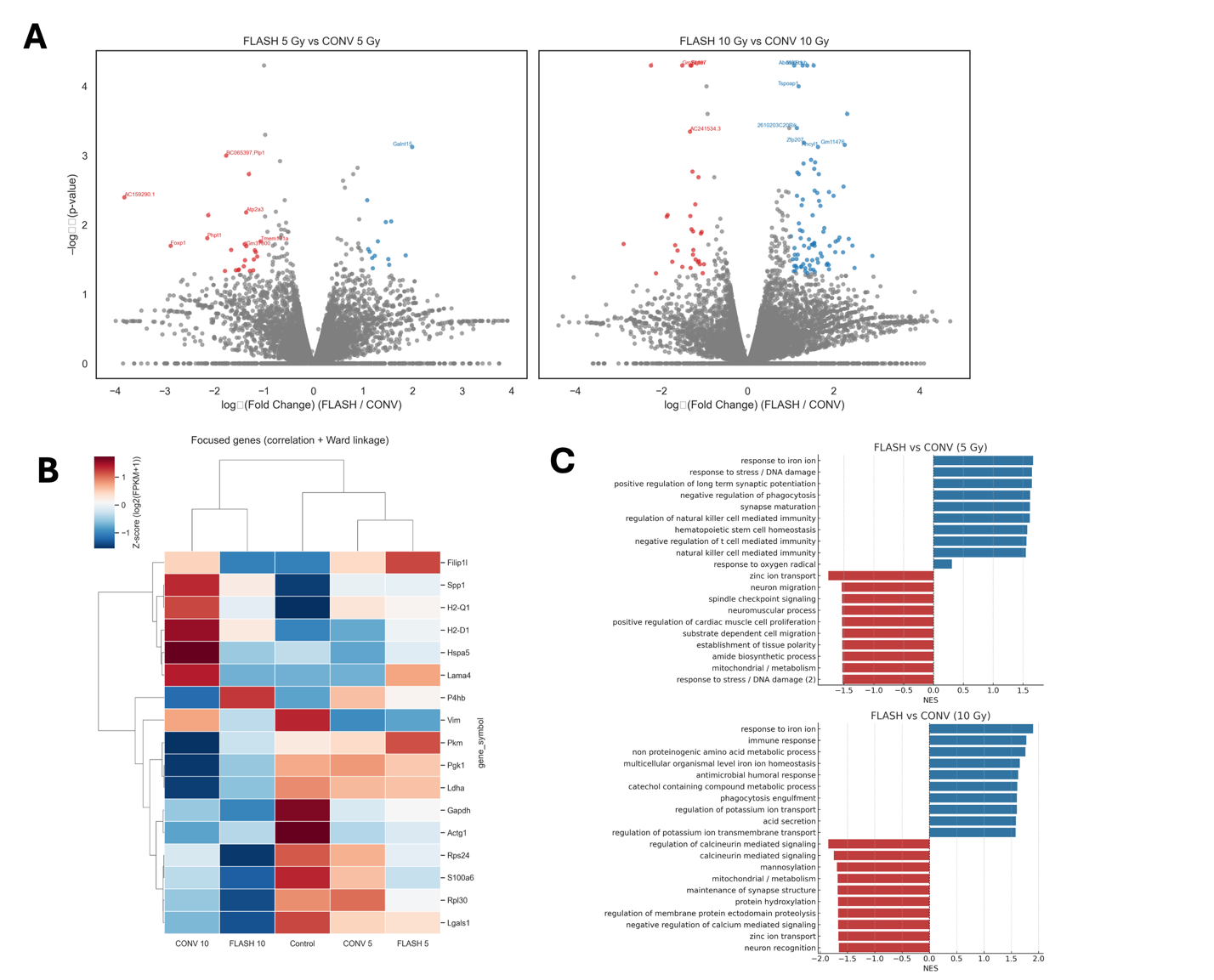


**Figure S3. (A)** Volcano plots showing differential gene expression between FLASH and CONV irradiation at 5 Gy and 10 Gy. Each point represents a gene (x-axis = log₂ fold change [FLASH/CONV], y-axis = –log₁₀ p value). Genes upregulated in FLASH (log₂ FC > 1, p < 0.05) are shown in red; genes upregulated in CONV (log₂ FC < –1, p < 0.05) are shown in blue; nonsignificant genes are grey.

Selected genes discussed in the text are labeled. The plots highlight the most significantly differentially expressed genes and illustrate consistent transcriptional shifts at both doses.

**(B)** Focused heatmap showing expression of selected genes across irradiation conditions following reclustering using correlation distance and Ward linkage. Heatmap displays z-scored log₂(FPKM+1) expression values for biologically relevant genes representing translation (Rps24, Rpl30), ER stress (Hspa5, P4hb), glycolysis (Gapdh, Pgk1, Pkm, Ldha), ECM and cytoskeletal remodelling (Spp1, Lama4, Vim, Filip1l, Actg1), and immune regulation (Lgals1, S100a6, H2-D1, H2-Q1). Rows and columns were clustered using correlation distance and Ward linkage to better reflect global sample relationships. The heatmap highlights dose-dependent divergence between FLASH and CONV irradiation at 10 Gy, with attenuated differences at 5 Gy.

**(C)** Gene set enrichment analysis (GSEA) of FLASH versus CONV irradiation at 5 Gy and 10 Gy. Normalised enrichment scores (NES) for the top positively and negatively enriched GO Biological Process pathways are shown for FLASH compared with CONV irradiation at 5 Gy (upper panel) and 10 Gy (lower panel). FLASH 10 Gy produced multiple significantly enriched pathways (FDR < 0.05), including translation, stress response, glycolysis, extracellular matrix organisation, and immune regulation. In contrast, enrichment patterns at 5 Gy showed similar directional trends but did not reach statistical significance, consistent with the weaker differential gene expression observed at this dose. NES values are shown for the top pathways ranked by enrichment magnitude.

**
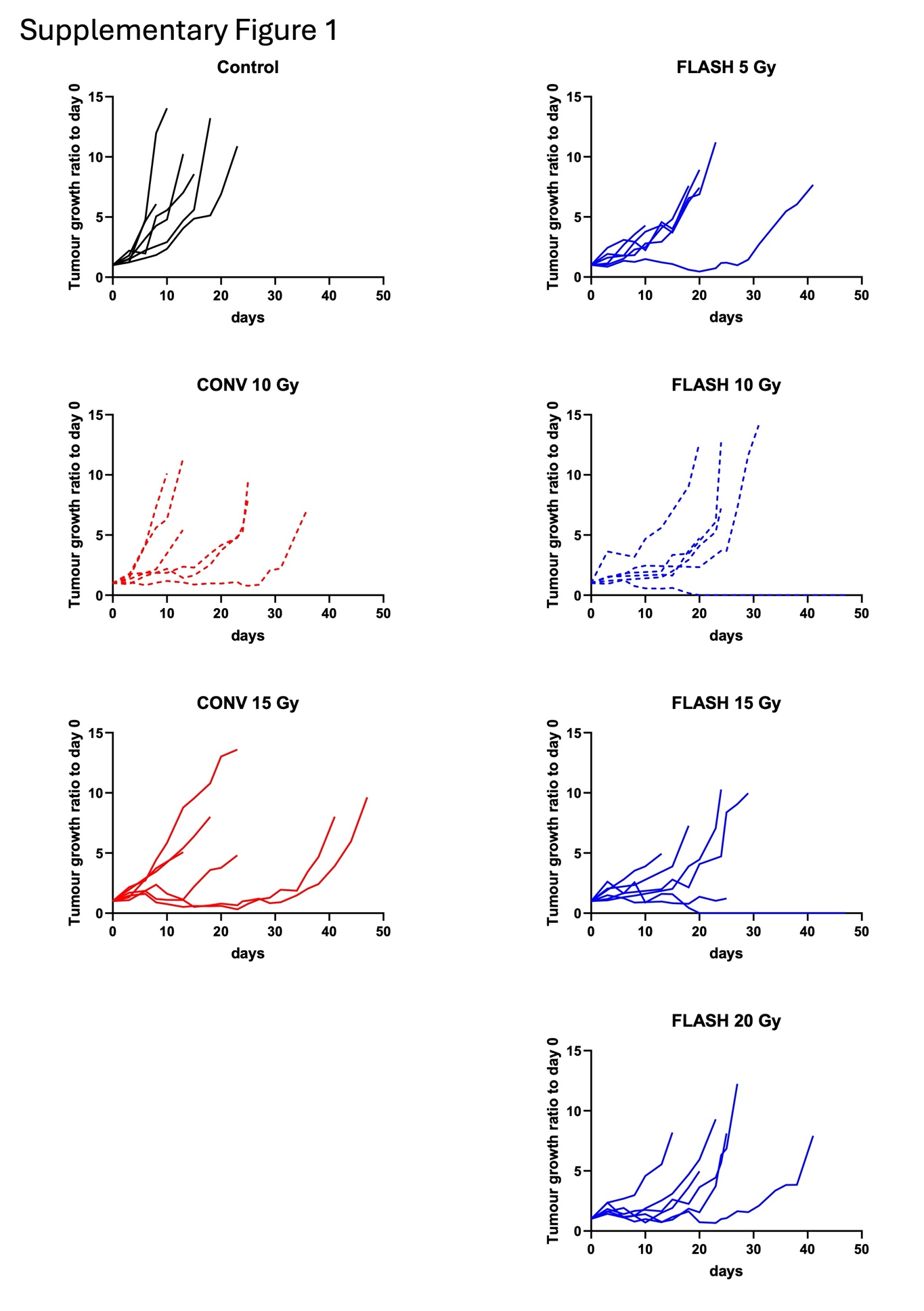
**

**Figure S4. Tumour growth delay of subcutaneous MBT2 tumour in C3H mice** **following specified doses of FLASH or CONV RT**, *n*=6 for each condition.


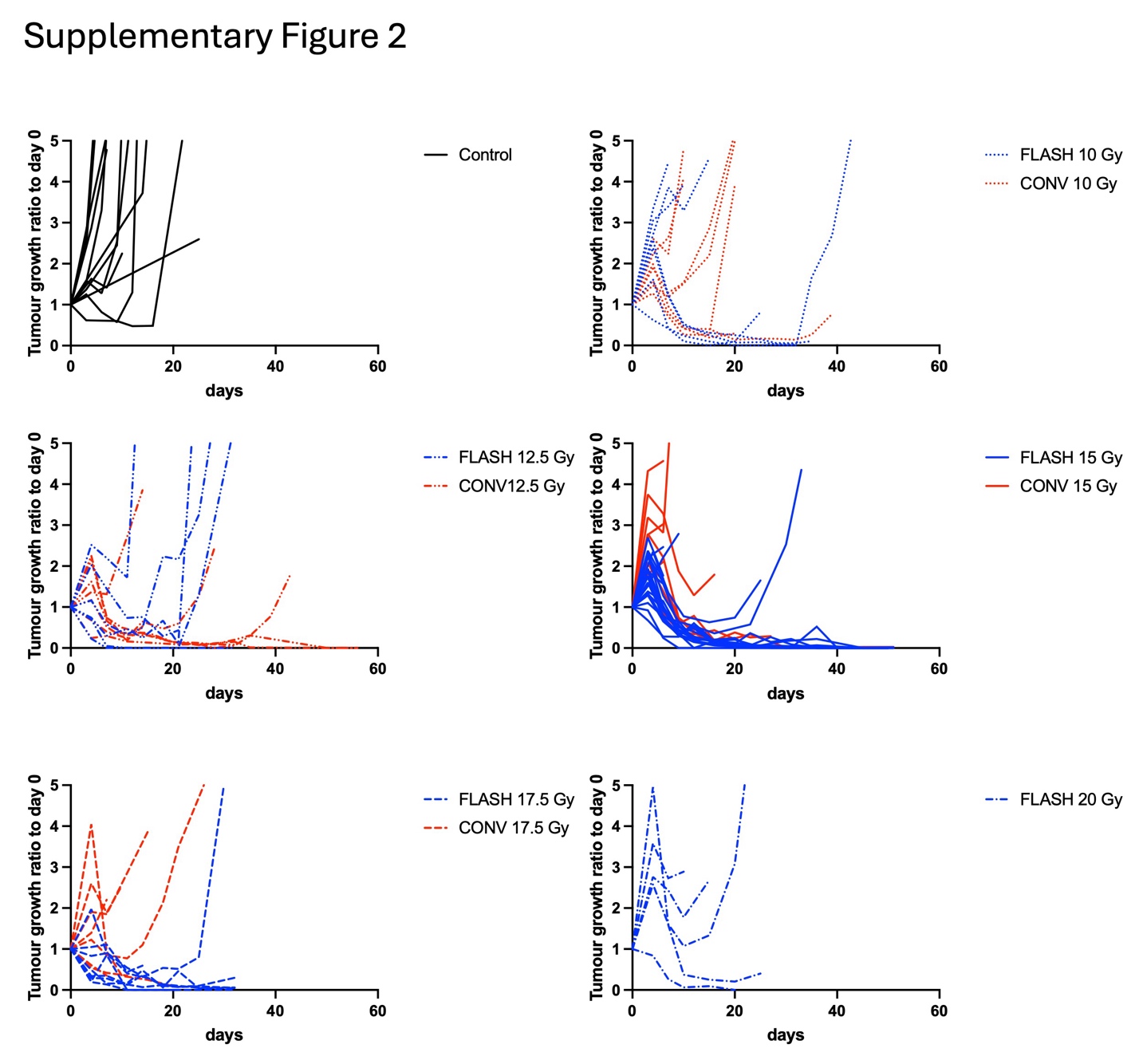


**Figure S5. Tumour growth for orthotopic MBT2 tumours in C3H mice following specified doses of CONV or FLASH RT.** Each line represents the tumour growth curve from one mouse, *n*= 5-20 for each condition.


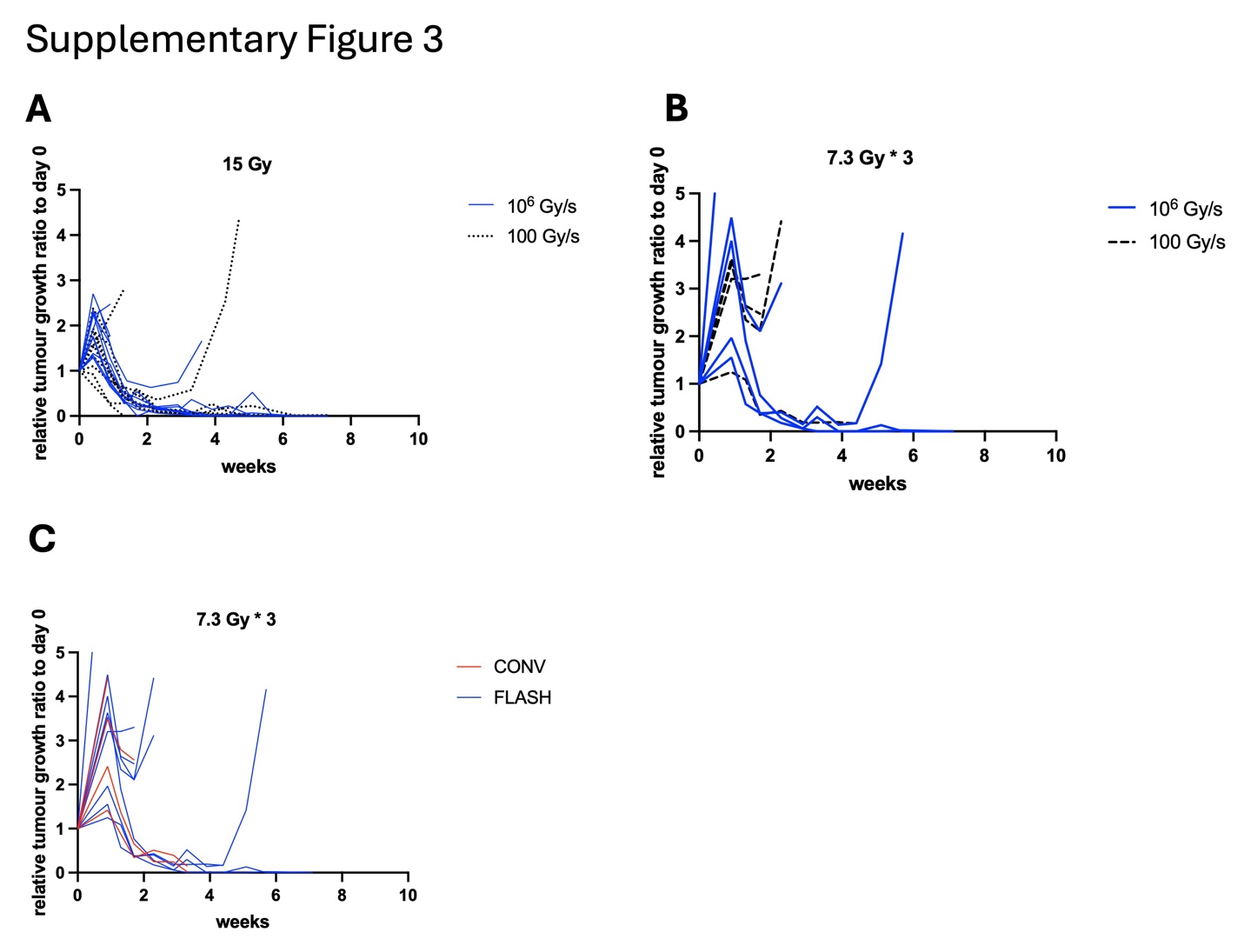


**Figure S6. Tumour growth curve for orthotopic MBT2 tumours on C3H mice following different fractionations.** A) single fraction FLASH RT at 15 Gy or B) (hypo-)fractionated treatment, and C) for CONV RT for a (hypo-)fractionated delivery.


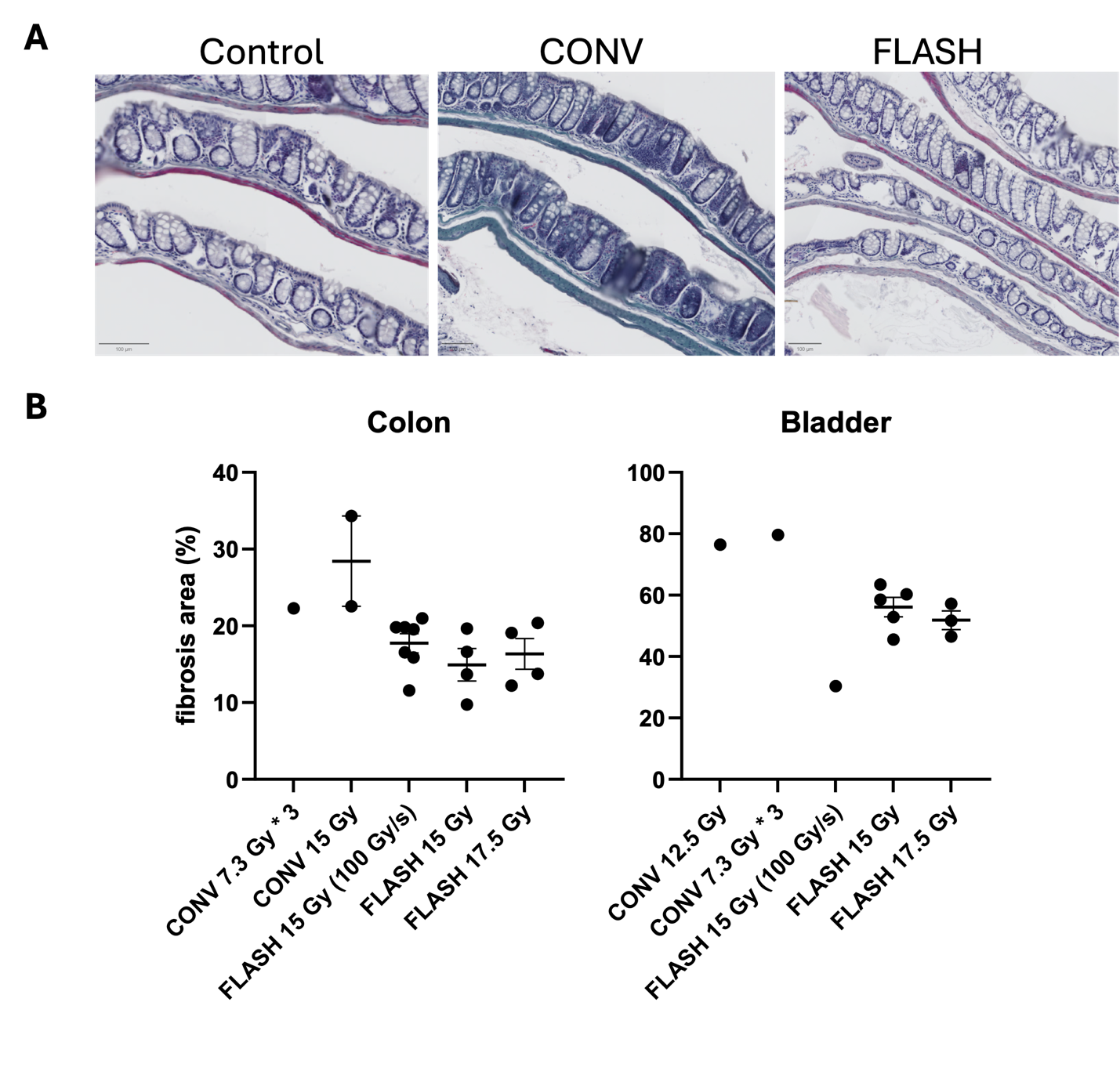


**Figure S7. Late tissue fibrosis following CONV or FLASH irradiation. (A)** Representative Masson’s trichrome–stained sections of colon harvested 30 weeks after 15 Gy irradiation, illustrating qualitatively reduced collagen deposition in FLASH-treated mice compared with CONV-treated controls. **(B)** Quantification of collagen-positive area for individual animals across treatment groups. Each point represents one animal; horizontal lines denote group means ± standard error of the mean.


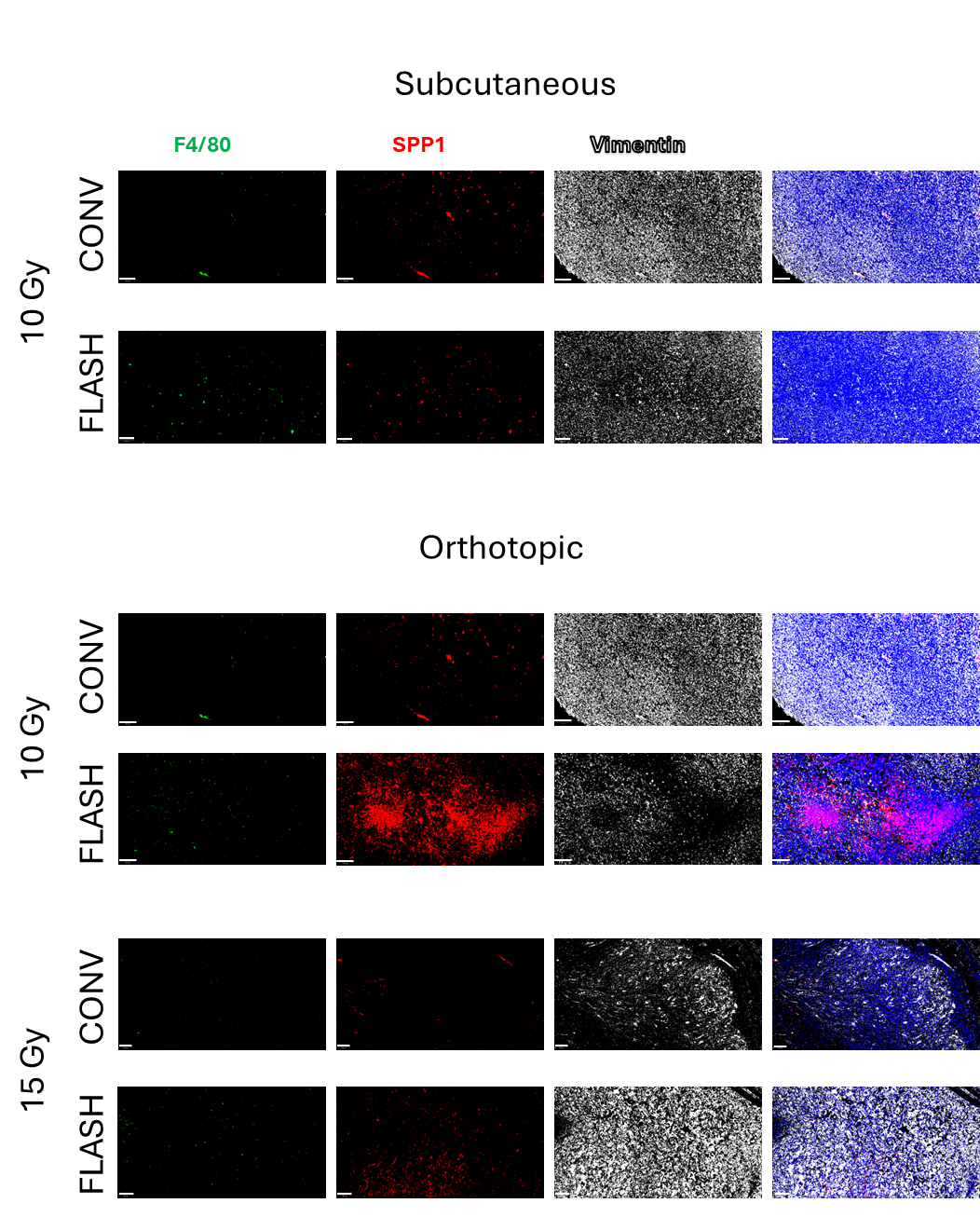


**Figure S8. Multiplex immunofluorescence of tumour microenvironment markers following CONV or FLASH irradiation.** Orthotopic tumour sections were stained for SPP1 (ECM remodelling, red), F4/80 (macrophages, green), vimentin (tumour-cell marker, white) and DAPI (nuclei, blue). Individual fluorescence channels are shown separately, followed by a merged image including DAPI. Images were acquired under identical exposure and gain settings. Staining patterns are presented qualitatively and were not subjected to quantitative image analysis. Scale bar shows 100 μm.

**Table S1. Beam parameters for conventional (CONV) and FLASH treatments.**

| **Treatment Modality** | | **Administered dose (Gy)** | **Dose rate**  **(Gy/s)** | **Delivery time (s)** | **Pulse repetition rate**  **(Hz)** | **Number of pulses**  **(n)** | **Dose-per-pulse**  **(mGy)** |  |
| --- | --- | --- | --- | --- | --- | --- | --- | --- |
| **CONV** | 5 | | ~10^-1^ | 50 | 25 | ≈1.25·10^3^ | ≈4 | |
|  | 10 | | ~10^-1^ | 100 | 25 | ≈2.5·10^3^ | ≈4 | |
|  | 12.5 | | ~10^-1^ | 125 | 25 | ≈3.1·10^3^ | ≈4 | |
|  | 15 | | ~10^-1^ | 150 | 25 | ≈3.75·10^3^ | ≈4 | |
|  | 17.5 | | ~10^-1^ | 175 | 25 | ≈4.38·10^3^ | ≈4 | |
|  | (3x) 7 | | ~10^-1^ | 70 | 25 | ≈1.75·10^3^ | ≈4 | |
| **FLASH** | 5 | | 1.47·10^6^ | 3.40·10^-6^ | n/a | 1 | 5.0·10^3^ | |
|  | 10 | | 3.00·10^3^ | 3.33·10^-3^ | 300 | 2 | 5.0·10^3^ | |
|  | 12.5 | | 1.88·10^3^ | 6.67·10^-3^ | 300 | 3 | 4.2·10^3^ | |
|  | 15 | | 1.00·10^2^ | 6.67·10^-3^ | 300 | 3 | 5.0·10^3^ | |
|  | 15 | | 2.25·10^3^ | 1.50·10^-1^ | 300 | 46 | 3.3·10^2^ | |
|  | 15 | | 4.41·10^6^ | 3.40·10^-6^ | n/a | 1 | 1.5·10^4^ | |
|  | 17.5 | | 2.63·10^3^ | 6.67·10^-3^ | 300 | 3 | 5.8·10^3^ | |
|  | 20 | | 2.00·10^3^ | 1.00·10^-2^ | 300 | 4 | 5.0·10^3^ | |
|  | (3x) 7 | | 1.00·10^2^ | 7.00·10^-2^ | 300 | 22 | 3.2·10^2^ | |
|  | (3x) 7 | | 2.06·10^6^ | 3.40·10^-6^ | n/a | 1 | 7.0·10^3^ | |
