# Supplementary figures and images for: FLASH radiotherapy enables dose escalation resulting in improved survival in an orthotopic muscle-invasive bladder cancer mouse model
Source: Br J Radiol. 2026 Mar 26;99(1182):1101–13. doi: 10.1093/bjr/tqag071 (PMC13273415; doi:10.1093/bjr/tqag071)

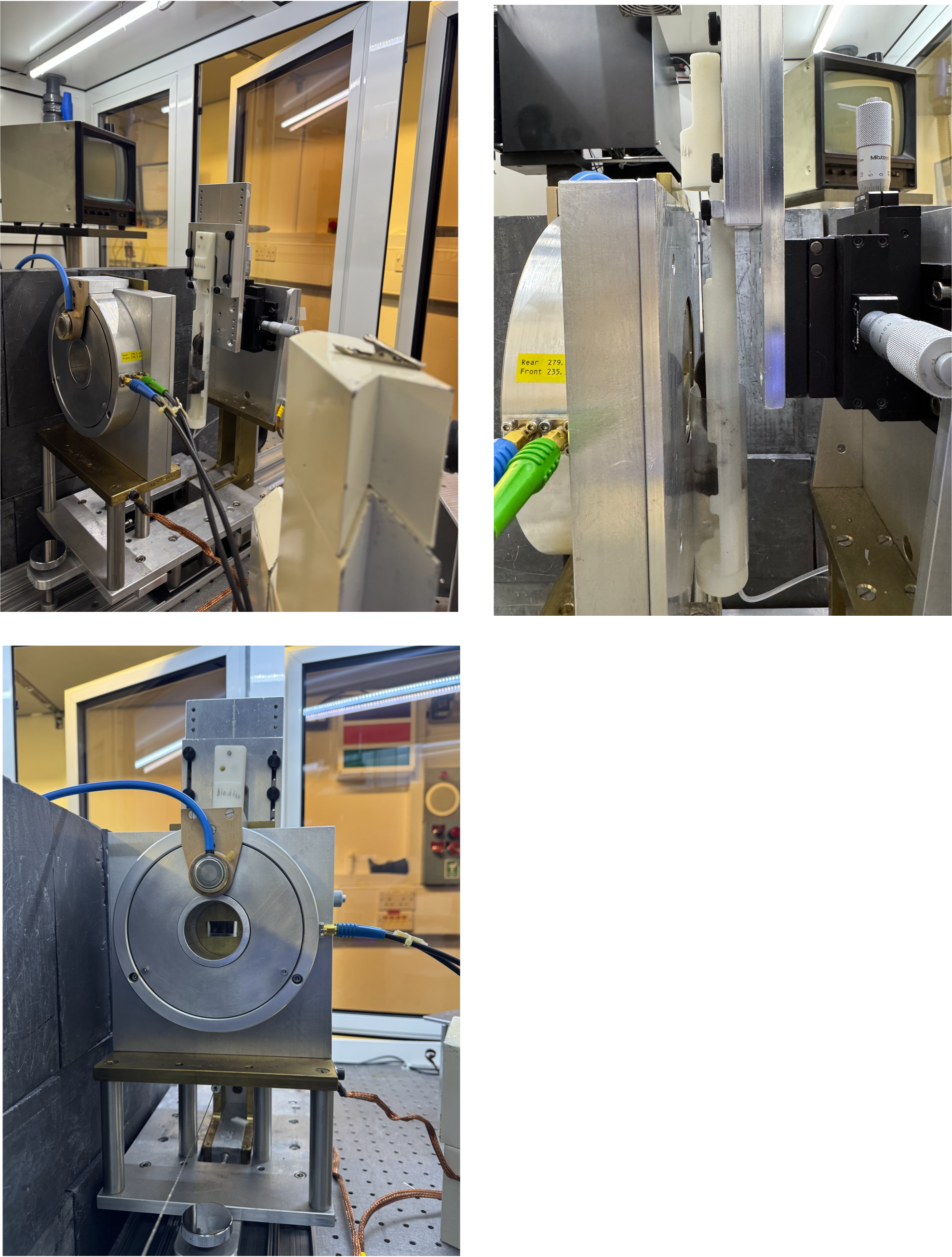

Supplement: tqag071_Supplementary_Data [file tqag071_supplementary_data.zip › Figure_S1.png]

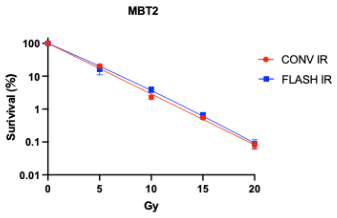

Supplement: tqag071_Supplementary_Data [file tqag071_supplementary_data.zip › Figure_S2.png]

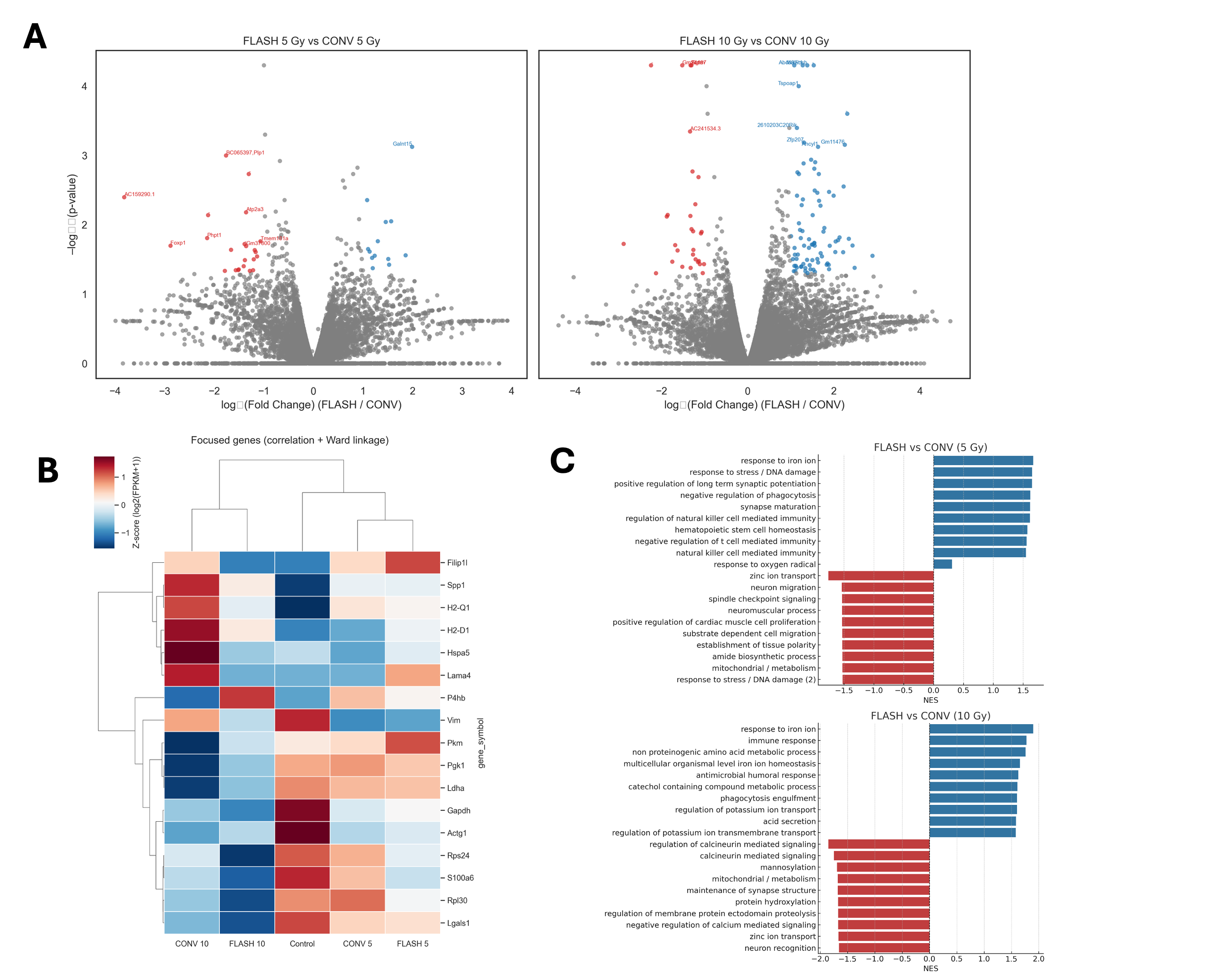

Supplement: tqag071_Supplementary_Data [file tqag071_supplementary_data.zip › Figure_S3.png]

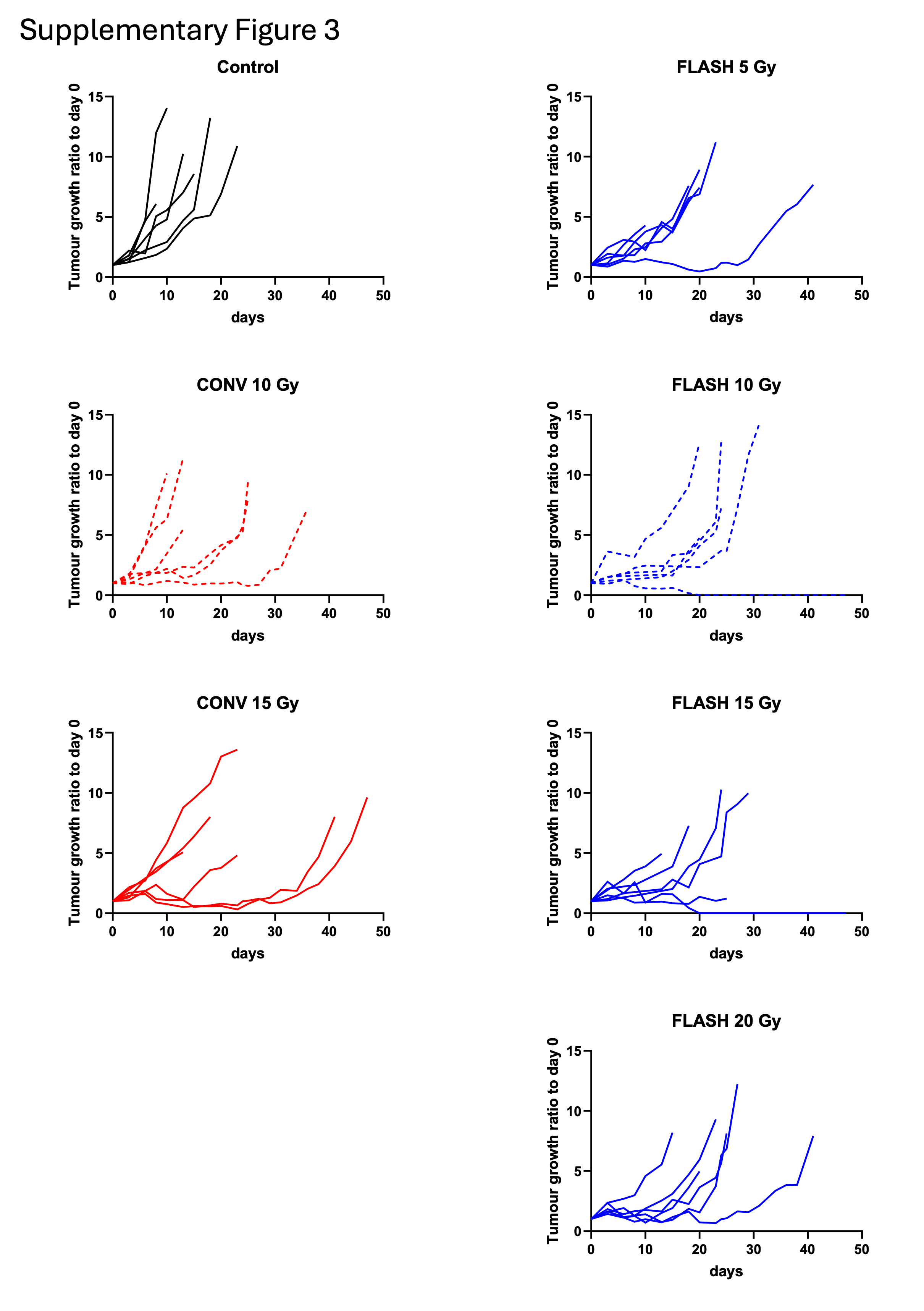

Supplement: tqag071_Supplementary_Data [file tqag071_supplementary_data.zip › Figure_S4.png]

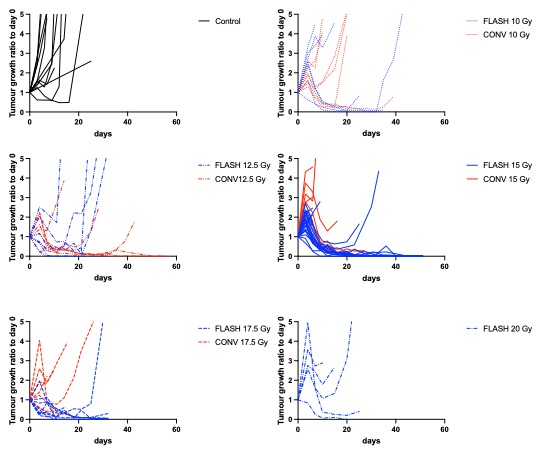

Supplement: tqag071_Supplementary_Data [file tqag071_supplementary_data.zip › Figure_S5.jpg]

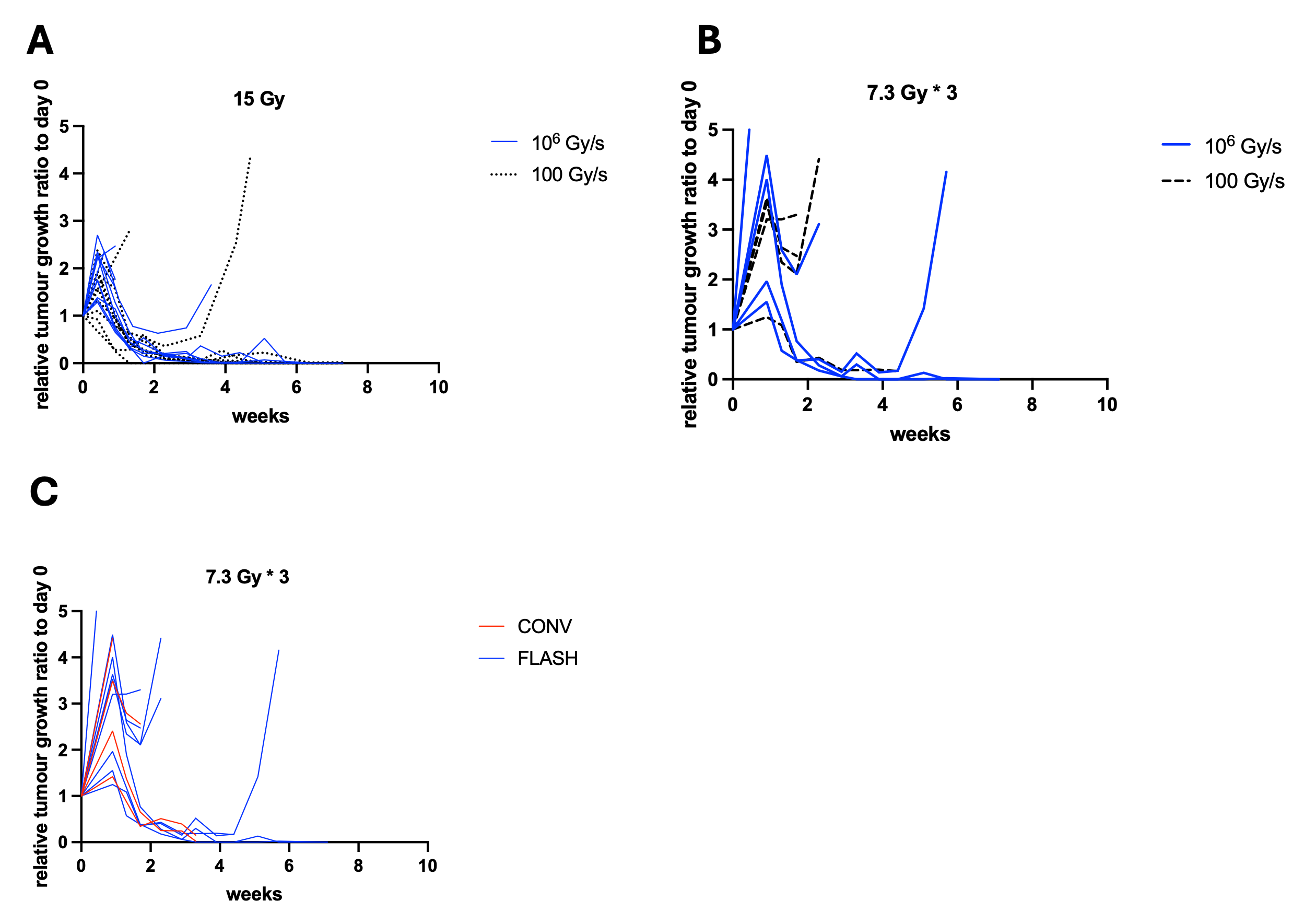

Supplement: tqag071_Supplementary_Data [file tqag071_supplementary_data.zip › Figure_S6.png]

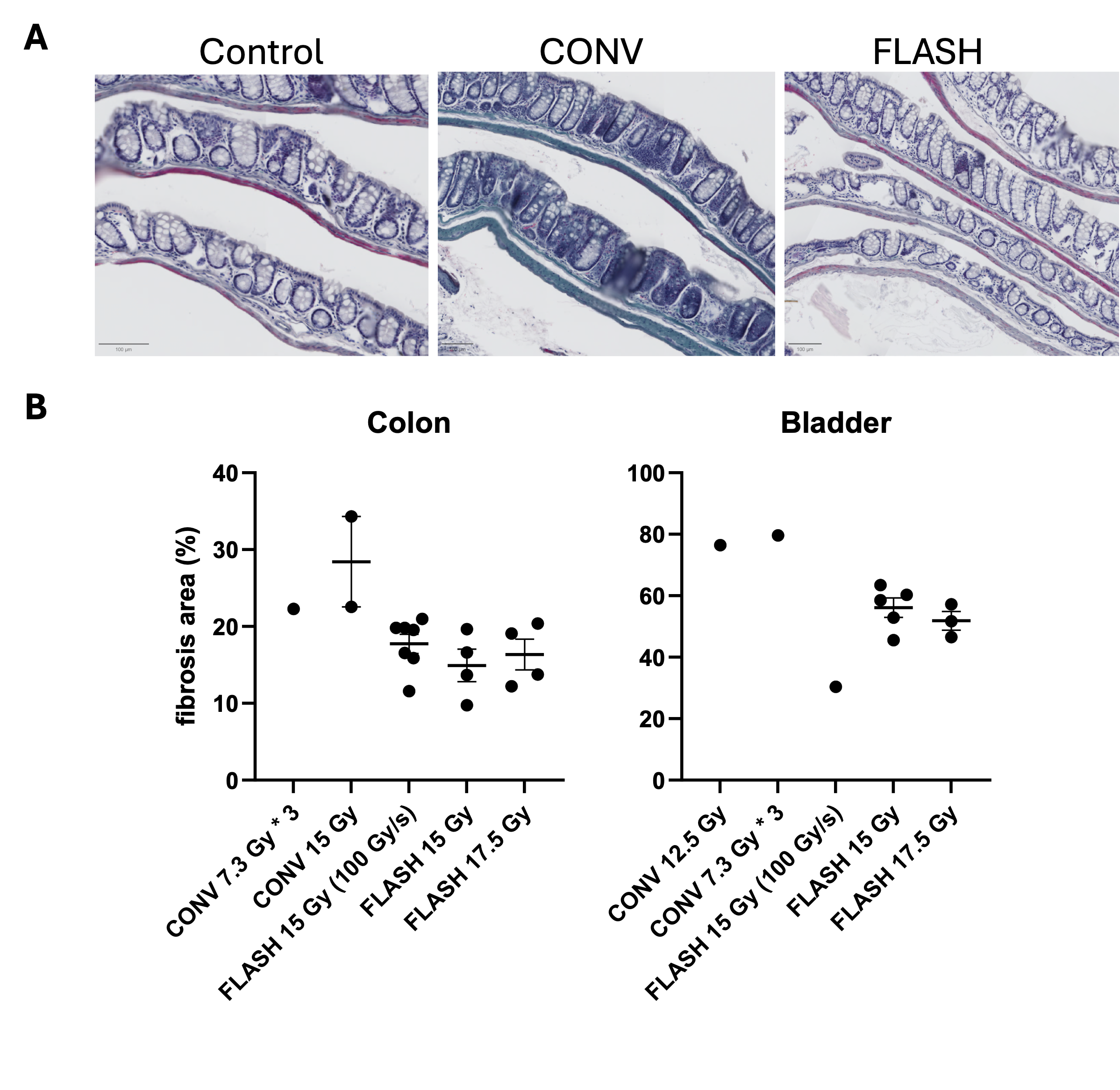

Supplement: tqag071_Supplementary_Data [file tqag071_supplementary_data.zip › Figure_S7.png]

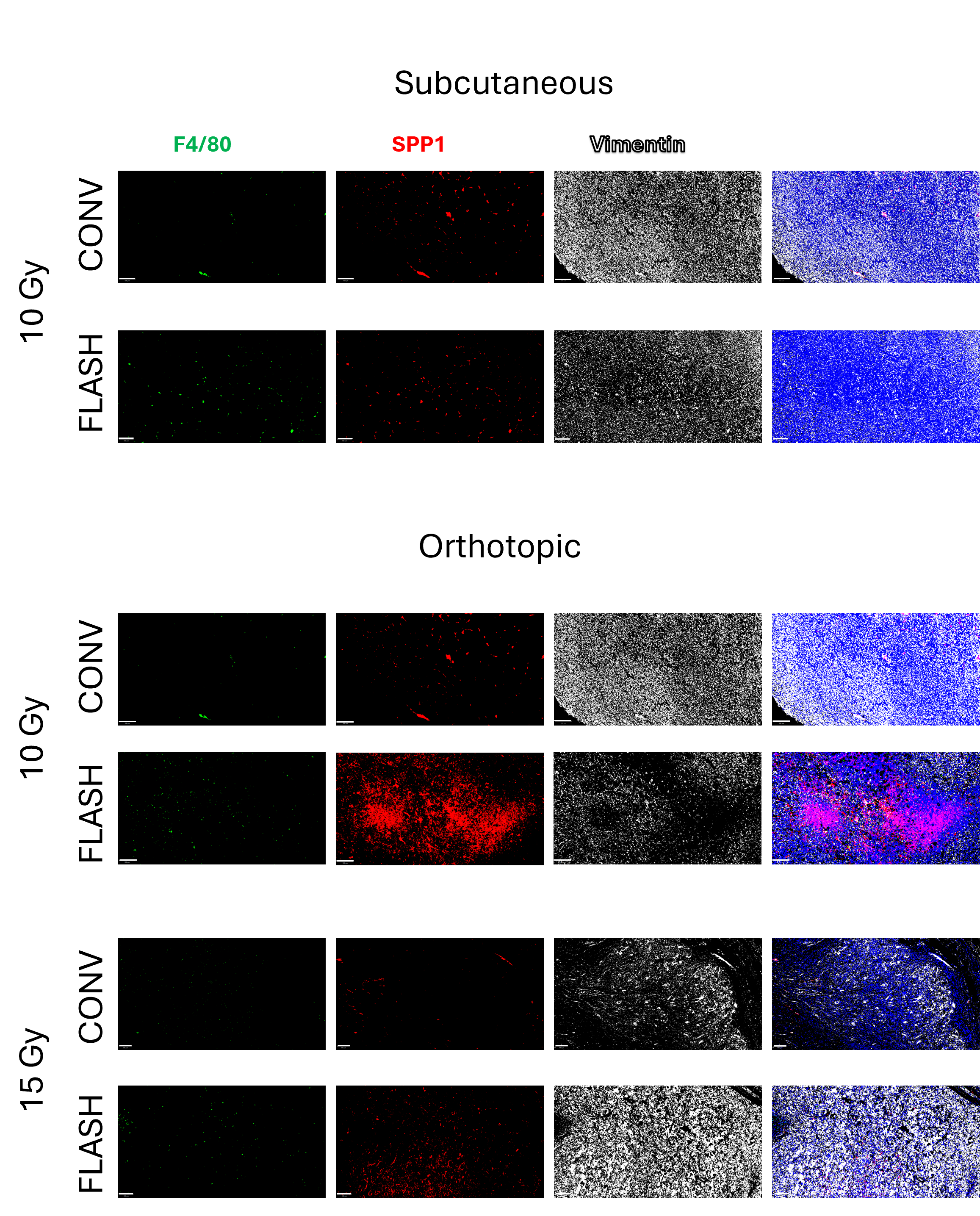

Supplement: tqag071_Supplementary_Data [file tqag071_supplementary_data.zip › Figure_S8.png]
